# Geometry-Driven Control of Linear and Nonlinear Optical Responses in *p*‑Nitroaniline: Insights into Structure–Property Relationships and Thermal Robustness

**DOI:** 10.1021/acsomega.5c08003

**Published:** 2025-12-04

**Authors:** Vinicius Manzoni, Rodrigo M. Gester, Antônio R. da Cunha, Gabriel I. Pagola, Guillermo F. Quinteiro Rosen, Patricio F. Provasi

**Affiliations:** † Instituto de Física, Universidade Federal de Alagoas, Maceio 57072-970 , AL, Brazil; ‡ Faculdade de Física, 422009 Universidade Federal do Sul e Sudeste do Pará, Maraba 68507-590, PL, Brazil; ¶ 37892 Universidade Federal do Maranhão, UFMA, Campus Balsas, Balsas CEP 65800-000, Maranhao, Brazil; § Facultad de Ciencias Exactas y Naturales, Departamento de Física, and CONICET−Universidad de Buenos Aires, Instituto de Física de Buenos Aires (IFIB), Universidad de Buenos Aires, Ciudad Universitaria, Buenos Aires 1428 , Argentina; ∥ Departamento de Física, Facultad de Ciencias Exactas y Naturales y Agrimensura, 28248 Universidad Nacional del Nordeste, IMIT-CONICET, Av. Libertad, Corrientes 5470 , Argentina; ⊥ Department of Physics, University of Northeastern, IMIT-CONICET, Av. Libertad, Corrientes 5500, Argentina

## Abstract

The nonlinear optical (NLO) response of organic chromophores is intrinsically governed by their molecular geometry; yet, the microscopic mechanisms that link specific deformations to optical properties remain unclear. Here, we reveal how controlled torsional and bond-length distortions in *p*-nitroaniline (pNA) dictate its dipole moment, polarizability, and hyperpolarizabilities, establishing clear geometry–property relationships. Using density functional theory and coupled-cluster calculations combined with thermal ensemble modeling, we uncover threshold-like behaviors: NH_2_ and NO_2_ torsions dominate the optical response, while local vibrational displacements have negligible effects. Remarkably, ensemble-averaged properties remain stable under thermally accessible fluctuations, demonstrating the intrinsic robustness of pNA’s NLO response. These results provide fundamental physical insight into geometry-driven modulation of optical properties, offering guiding principles for the rational design of molecular photonic materials.

## Graphical abstract

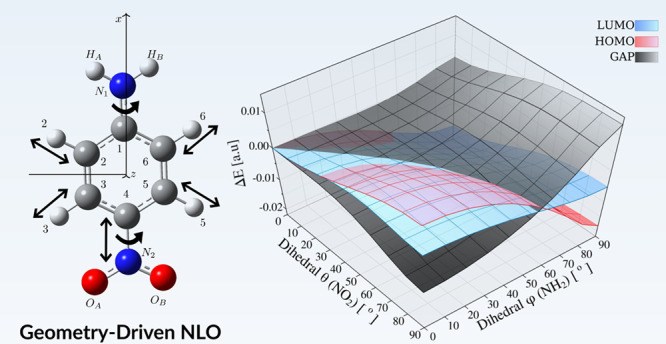

## Introduction

Nonlinear optical (NLO) responses are susceptible to molecular geometry and electronic structure variations.^1,2^ However, the fundamental mechanisms linking specific molecular deformations to their linear and NLO responses remain incompletely understood, particularly in prototypical push–pull chromophores. Investigating *p*-nitroaniline (pNA, C_6_H_6_N_2_O_2_) under torsional, contractional, and stretching deformations provides an opportunity to reveal geometry–property relationships and threshold-like behaviors that dictate NLO performance, thereby offering direct insight into structure–property relationships relevant to molecular NLO behavior. These geometric modifications can significantly redistribute electron density and modulate both linear and NLO responses, offering guidance for the rational design of photonic and optoelectronic materials and providing new physical insight into the intrinsic robustness and tunability of molecular NLO properties.

For instance, torsional movements, such as the twisting of the nitro (−NO_2_) or amino (−NH_2_) groups relative to the benzene ring, can disrupt the conjugation pathway that facilitates charge transfer. This disruption underlies the observed threshold behavior, where moderate torsions induce negligible effects, but larger rotations trigger abrupt changes in dipole moment and hyperpolarizability (β). Similarly, contractional or stretching movements along specific bonds (e.g., C–N or C–H) can modify bond lengths and angles, thereby influencing the polarizability and resonance characteristics of pNA. Moreover, identifying which distortions meaningfully affect the electronic distribution and which leave it robust provides a clear physical basis for NLO property tuning, a concept that can be generalized to other push–pull chromophores and organic NLO dyes.

Based on this understanding of the relationship between molecular geometry and optical response, studies are often conducted using computational methods such as time-dependent density functional theory (TD-DFT), which allows the simulation of structural perturbations and the prediction of their effects on NLO properties.^3^ Experimental techniques, including vibrational spectroscopy and dynamic hyper-Rayleigh scattering (HRS), complement these calculations by providing insight into how molecular vibrations and deformations correlate with observed NLO behavior.^4,5^ However, most previous computational studies have not systematically separated the contributions of localized vibrations from large-amplitude torsional distortions, leaving a gap in the mechanistic understanding of geometry-driven optical modulation.

Chromophores with NLO response exhibit changes in their nonlinear response when subjected to an external electric field or immersed in a solvent medium.^6–12^ These effects are often linked to conformational changes in the molecular structure, which influence linear and NLO responses. Isolating the intrinsic geometrical contributions, independent of solvent or field effects, provides new insight into the fundamental structure–property relationship that governs the NLO behavior.

Several studies have investigated the influence of solvents and external electrostatic fields on the NLO behavior of chromophores.^9–17^ It is well established that the solvent environment can significantly change the molecular polarizabilities and hyperpolarizabilities. For instance, Damasceno et al.^18^ showed that solvation enhances the nonlinear response due to electronic stabilization. More recently, Quinteiro Rosen et al.^19^ showed that, in the case of imidazole and pyrrole, the influence of inhomogeneous electric fields on NLO properties is generally less pronounced than the effects induced by solvation. Here, we complement these studies by demonstrating how pNA’s optical properties are inherently robust under thermally accessible distortions, a feature critical to understanding its gas-phase baseline behavior before environmental effects are introduced.

From a molecular structure perspective, pNA belongs to a class of common organic second-order NLO compounds with push–pull behavior, composed of donor (D) and acceptor (A) groups connected by a π-conjugated bridge.^20–22^ This molecular arrangement ensures the high susceptibility of the electron density to external electric fields while maintaining molecular asymmetry. pNA plays an essential role as the prototype of the D−π–A model, widely studied using both experimental techniques^23–27^ and theoretical methods.^28–32^ Its NLO properties have been characterized in the gas phase,^33,34^ in solution,^35–39^ and in bulk structures, including molecular crystals,^40^ heterogeneous environments,^31^ and polymeric nanofibers.^25,41^

Based on the premise that such structural modifications in pNA can serve as a model for understanding how geometry affects NLO responses, this study focuses on the theoretical investigation of the NLO properties of various pNA rotational and vibrational isomers in the gas phase. This approach allows us to (i) isolate the intrinsic geometric effects from environmental perturbations, (ii) identify threshold-like behaviors that dictate linear and nonlinear responses, and (iii) assess the thermal robustness of these properties via ensemble modeling, providing clear physical insight for the rational design of molecular photonic materials.

The main objective of the present work is to explore how specific geometric changes influence the dipole moment, energy, and NLO properties of pNA. By systematically probing torsional and bond-length distortions, we aim to reveal threshold-like behaviors and intrinsic structure–property relationships that govern optical responses. This analysis aims to clarify the dependence of these properties on the molecular geometry, a relationship that is not straightforward to infer. In this context, the stretching and contraction processes simulate variations, such as isotope substitution or vibrational displacements related to temperature or normal modes. Rotational deformations, in turn, mimic different spatial configurations that the molecule might adopt when in thermal equilibrium at temperature *T*, allowing us to connect static geometric effects to ensemble-averaged thermal behavior and provide new physical insight into the robustness and tunability of molecular NLO properties.

## Theoretical Details

### Structural Modifications Considered

A systematic approach was adopted to investigate how specific structural modifications impact the optical properties of pNA. Several such changes and their combinations were considered for the analysis, as illustrated in Figure 1. Specifically, the following structural modifications were systematically considered: (i) rotation of the NH_2_ group around the main molecular axis (about φ ≡ τ­(HA–N1–C1–C2)); (ii) rotation of the NO_2_ group around the same axis (about θ ≡ τ­(OA–N2–C4–C3)); (iii) stretching and contraction of the C4–N2 bond; (iv) independent and combined stretching/contraction of the H2 and H3 bonds with H6; and (v) simultaneous rotation of NH_2_ with stretching or contraction of the C3–H3 bond. Each molecular geometry was manually modified according to the structural patterns defined in Figure 1 before calculating the property of interest.

**Figure 1:**
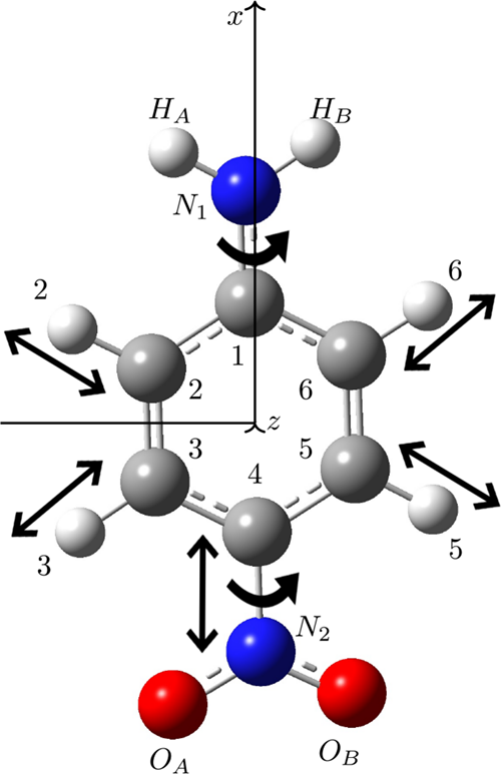
Molecular structure of *p*-nitroaniline (C_6_H_6_N_2_O_2_) with relevant atomic positions. The illustrated arrows indicate the rotations, bond stretches, and contractions analyzed in this study to evaluate their impact on linear and nonlinear optical (NLO) properties.

We note that this systematic structural analysis is not intended to replace a full molecular dynamics treatment. Rather, it provides a controlled framework to probe how specific distortions affect the electronic structure. In particular, the NH_2_ and NO_2_ torsional coordinates correspond to low-frequency vibrational modes (see Table S2, SI), which are thermally accessible and therefore expected to contribute significantly to the conformational ensemble. This supports the relevance of the structural modifications considered in this work.

### Computational Details

All quantum chemical calculations were performed using the Dalton software package.^42^ The B3LYP functional^43–45^ was employed in conjunction with the 6-311++G** basis set^46^ to compute the dipole moment, the energies of the frontier molecular orbitals (HOMO and LUMO), and the NLO properties: polarizability (α), first hyperpolarizability (β), and second hyperpolarizability (γ). All hyperpolarizability tensors were computed at a frequency of 0.043 au, corresponding to a fundamental wavelength of approximately 1064 nm, consistent with typical Nd:YAG laser experiments in THG and HRS measurements. All calculations were performed without imposing symmetry constraints. Also, all calculated values were expressed in atomic units (au) and normalized to their gas-phase equilibrium values (reported in Table 1 – line 1) to facilitate the comparison of how each property responds to each specific structural modification. Consequently, the plotted values should be interpreted as relative deviations centered around zero, enabling visualization of structural effects. All potential-energy and property maps were obtained from rigid scans performed on the optimized gas-phase geometry. For each map, the selected dihedral or bond coordinate was systematically varied, while all remaining internal coordinates were kept fixed. No further geometry optimization was carried out at each point.

**Table 1:** Nonlinear Optical Properties, Electric Dipole, Frontier Orbitals, and Energy Gap (in au) of pNA in the Gas Phase Calculated at Different Levels of Theory

property	DFT	CCS^b^	CC2^b^	CCSD^b^
μ	3.087	3.090	2.770	2.827
LUMO	–0.0857			0.0516^a^
HOMO	–0.2371			–0.3236^a^
gap	0.1514			0.3752^a^
α	104	99	106	100
β	1372.7	586.5	1139.4	922.6
γ	215,826			

a HOMO, LUMO and gap computed at the Hartree–Fock level.

b Calculations performed with the 6-311++G** basis set.

### Reference Data and Method Comparison

Table 1 shows gas-phase equilibrium values of all calculated properties using DFT. Also, the results obtained at the CCSD/6-311++G** level of theory that serve as a reference for assessing the reliability of the B3LYP results are shown. The dipole moment and polarizability (α) calculated at the DFT level agree well with the CCSD values. The first hyperpolarizability (β) is consistently overestimated by approximately a factor of 2 relative to CCSD, though it remains within the same order of magnitude. Due to the prohibitive computational cost, CCSD-level calculations of the second hyperpolarizability (γ) were not feasible.

Table 1 also shows the same properties calculated in the gas phase but at another level of calculation to validate the B3LYP/6-311++G** results. Thus, taking the CCSD/6-311+G** calculations as the reference value, the DFT estimation is considerably good up to polarizability α. The first hyperpolarizability β is overestimated by approximately a factor of 2 relative to CCSD, although it remains on the same order of magnitude. For the second hyperpolarizability γ, no CCSD-level calculations were feasible due to computational limitations. Nevertheless, the CCS and CC2 results were included to illustrate the evolution of correlation effects and to provide an intermediate reference between the DFT and CCSD levels, supporting the robustness of the overall trends.

### NLO Properties

As electrical linear and NLO parameters are the focus of this investigation, we dedicate a few lines to mention that these effects arise when the light interacts with the matter. In such a case, the energy of the system can be expanded in a Taylor series like:

$$E(F)\;=\;E(0)\;-\;\mu_{i}F_{i}\;-\;\frac{1}{2!}\alpha_{ij}F_{i}F_{j}\;-\;\frac{1}{3!}\beta_{ijk}F_{i}F_{j}F_{k}\;-\;\frac{1}{4!}\gamma_{ijkl}F_{i}F_{j}F_{k}F_{l}\;-\cdots$$ 1

in which μ is the permanent dipole moment, α is the dipole polarizability, a tensor of rank 2, whose diagonal components might be combined to give the isotropic contribution (α_iso_)­

$$\alpha_{\mathrm{iso}}\;=\frac{\;1}{3}\;(\alpha_{xx}\;+\;\alpha_{yy}\;+\;\alpha_{zz})$$ 2

The isotropic polarizability can be used to infer the refractive index (*n*) using the Lorentz–Lorenz equation^47,48^

$$\frac{n^{2}\;-\;1}{n^{2}\;+\;2}\;=\;\frac{4\pi \alpha_{\mathrm{iso}}}{3V_{\mathrm{mol}}}$$ 3

where *V*_mol_ is the molecular volume.

Concerning the first hyperpolarizability (β), it is a 3 × 3 × 3 tensor with 27 components, and in the presence of frequency-dependent light, this quantity is better described by the HRS apparatus^49,50^ to give the frequency-dependent first hyperpolarizability as

$$\beta_{\mathrm{HRS}}\;=\;\sqrt{\frac{10}{45}|\beta_{J=1}{|}^{2}+\frac{10}{105}|\beta_{J=3}{|}^{2}}$$ 4

In this equation, β_*J*=1_ and β_*J*=3_ correspond to the dipolar and octupolar tensor, respectively, written like

$$|\beta_{j=1}{|}^{2}\;=\;\frac{3}{5}\sum_{i}^{x,y,z}\beta_{iii}^{2}\;+\;\frac{6}{5}\sum_{i\neq j}^{x,y,z}\beta_{iii}\beta_{ijj}\;+\;\frac{3}{5}\sum_{i\neq j}^{x,y,z}\beta_{jii}^{2}\;+\;\frac{3}{5}\sum_{i\neq j\neq k}^{x,y,z}\beta_{ijj}\beta_{ikk}$$ 5

and

$$|\beta_{j=3}{|}^{2}\;=\;\frac{2}{5}\sum_{i}^{x,y,z}\beta_{iii}^{2}\;-\;\frac{6}{5}\sum_{i\neq j}^{x,y,z}\beta_{iii}\beta_{ijj}\;+\;\frac{12}{5}\sum_{i\neq j}^{x,y,z}\beta_{jii}^{2}\;-\;\frac{3}{5}\sum_{i\neq j\neq k}^{x,y,z}\beta_{ijj}\beta_{ikk}\;+\;\sum_{i\neq j\neq k}^{x,y,z}\beta_{ijk}^{2}$$ 6

The above-described tensors can be combined to give the anisotropy factor $\rho \;=\;\frac{|\beta_{J=3}{|}^{2}}{|\beta_{J=1}{|}^{2}}$ , which defines the dipolar $\left(\varphi_{J=1}\;=\;\frac{1}{1\;+\;\rho }\right)$ , and octupolar $\left(\varphi_{J=3}\;=\;\frac{\rho }{1\;+\;\rho }\right)$ contributions. Alternatively, it can be discussed the dipolar-octupolar relationship introducing the concept of depolarization ratio, $\mathrm{DR}\;=\;\frac{\left\langle \beta_{ZZZ}^{2}\right\rangle }{\left\langle \beta_{ZXX}^{2}\right\rangle }$ ,^49,50^ which might be obtained by manipulating the tensorial components of β_HRS_.

The second hyperpolarizability (γ) is the last parameter of interest. It is responsible for the third-harmonic generation effect. This one is a fourth-rank tensor (3 × 3 × 3 × 3) with 81 components. Fortunately, within the electric-field-induced second-harmonic generation setup, the middle indices can be interchanged γ*_ijkl_* = γ_*ikjl*_. Such simplification allows us to work with only 15 components, and the third harmonic generation γ­(−3ω;ω, ω, 0) = γ_THG_ is given as^51^

$$\gamma_{\mathrm{THG}}\;=\;\frac{1}{5}[\gamma_{xxxx}\;+\;\gamma_{yyyy}\;+\;\gamma_{zzzz}\;+\;2(\gamma_{xxyy}\;+\;\gamma_{xxzz}\;+\;\gamma_{yyzz})]$$ 7

Together with the other magnitudes described, they are represented hereafter in the corresponding figures. In Table 1 and throughout the text, the symbols α, β, and γ denote the isotropic mean polarizability (α_iso_), the hyper-Rayleigh scattering first hyperpolarizability (β_HRS_), and the third-harmonic generation second hyperpolarizability (γ_THG_), respectively.

## Results and Discussion

The NLO response of organic molecules is sensitive to structural and electronic factors. In this study, a systematic investigation was performed to evaluate how specific geometric modifications, such as bond stretching, contraction, and rotational deformations, change the dipole moment, frontier orbital energies (HOMO and LUMO), polarizability (α), and first and second hyperpolarizabilities (β and γ) of *p*-nitroaniline (pNA). The relevance of such an approach is supported by recent theoretical results showing that geometry-dependent mechanisms play a central role in determining electronic and optical behavior.^2,52^ In particular, these works demonstrated that small structural changes in merocyanine dyes can have a decisive impact on linear and NLO responses. These effects often compete with or even outweigh contributions from external polarization fields or solvent interactions, highlighting the fundamental role of intrinsic geometry in modulating properties such as hyperpolarizability and solvatochromism. These studies underline the importance of isolating geometric contributions when analyzing linear and NLO responses, especially under realistic conditions where external stimuli (e.g., electric fields, solvent polarity, or thermal fluctuations) may induce structural flexibility.

Here, the structural distortions examined represent physically plausible perturbations, such as those induced by thermal fluctuations or local environmental interactions. The results are presented in terms of relative variations normalized to gas-phase reference values, highlighting the robustness of the property trends in response to geometrical changes. Particular attention is given to threshold-like behaviors, in which certain properties remain nearly invariant up to a critical deformation before exhibiting abrupt nonlinear shifts. These patterns offer insights into the geometric sensitivity of optical properties and suggest structural design strategies for tailoring the NLO performance.

### Electronic Structure and Resonance Analysis of pNA

The NLO response of *p*-nitroaniline arises directly from its push–pull character in which the electron-donating NH_2_ group and the electron-withdrawing NO_2_ group are conjugated through the aromatic π-system. This framework can be qualitatively understood in terms of two limiting resonance forms: (i) a neutral structure in which the amino and nitro substituents retain localized charges and (ii) a charge-separated form that illustrates intramolecular electron transfer from the donor through the π-conjugated ring toward the acceptor. These resonance contributors provide an intuitive picture of the electronic redistribution that underlies the large dipole moment and hyperpolarizability of pNA.

To complement this qualitative resonance picture, we analyzed the frontier molecular orbitals of pNA (Figure 2). At equilibrium planar geometry, the HOMO is mainly localized on the amino donor and delocalized over the adjacent π-system of the aromatic ring, whereas the LUMO is centered on the nitro acceptor. This orbital distribution corresponds directly to the charge-separated resonance contributor, confirming the donor–acceptor charge-transfer nature of the excitation. Upon torsional deformation of the NH_2_ or NO_2_ groups, the overlap between the donor-centered HOMO and the acceptor-centered LUMO progressively decreases, weakening conjugation and shifting the electronic structure toward the neutral resonance form. Natural bond orbital (NBO) charges further support this interpretation (Table S1 in the Supporting Information). In the planar geometry, the nitro oxygens carry a substantial negative charge (−0.40 *e* each), indicating efficient electron donation from the amino group through the aromatic bridge. Upon torsional deformation, these oxygens become less negative (≈ −0.35 *e*), revealing a reduction in charge transfer efficiency. The amino nitrogen charge remains nearly constant, but the diminished accumulation on the acceptor atoms confirms that planarity maximizes the ground-state push–pull character, whereas torsion suppresses it.

**Figure 2:**
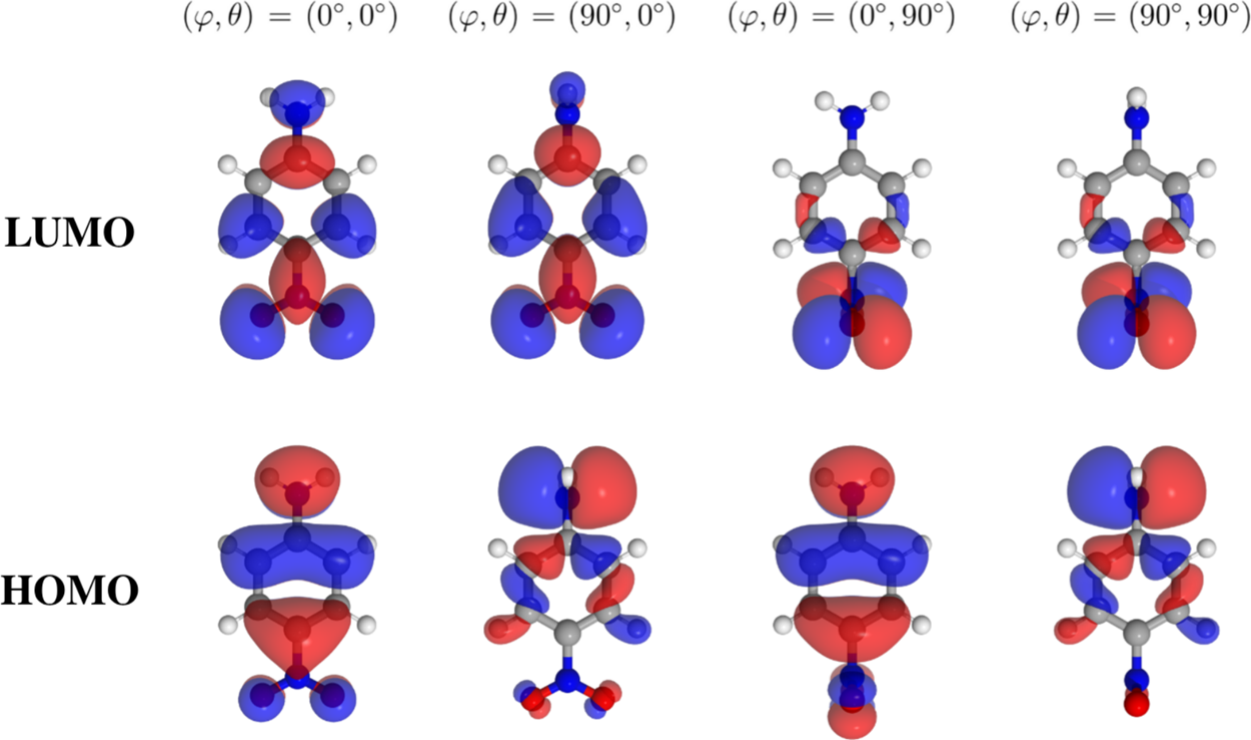
Frontier molecular orbitals (HOMO and LUMO) of *p*-nitroaniline at selected torsional angles of the amino (φ) and nitro (θ) groups. At the planar geometry (φ, θ = 0°, 0°), the HOMO is mainly localized on the donor NH_2_ group and the aromatic π-system, while the LUMO is centered on the NO_2_ acceptor, consistent with intramolecular charge transfer. Increasing torsion reduces the overlap between donor- and acceptor-centered orbitals, thereby weakening the push–pull character.

In addition, we analyzed the vibrational spectrum to connect the torsional deformations to ground-state dynamics. For clarity, we denote the NH_2_ torsion by φ and NO_2_ by θ. The lowest-frequency modes correspond to the torsions of the NO_2_ (63 cm^–1^, θ) and NH_2_ (371 cm^–1^, φ) groups, as illustrated in the computed IR spectrum (Figure S18) and summarized in Table S2 (SI). Although these modes exhibit weak IR intensities, their low frequencies make them thermally accessible, and thus, they are expected to contribute to modulating conjugation and the donor–acceptor push–pull character of pNA.

### Dipole Moment

We begin by analyzing the dipole moment response to the rotation of the NH_2_ and NO_2_ groups around the main molecular axis. In this analysis, each group was rigidly rotated from 0 to 90° in increments of 10°, while all other internal coordinates were kept fixed. The resulting variation in dipole moment, computed relative to the gas-phase equilibrium value, is shown in Figure 3, with the NH_2_ and NO_2_ results presented in the left and right panels, respectively. The rotation was performed about the dihedral angles HA–N1–C1–C2 for NH_2_ and OA–N2–C4–C3 for NO_2_ (hereafter denoted by φ and θ, respectively), with positive angles defined as anticlockwise rotations viewed along the N → C (ring) bond. The rotation direction (clockwise or counterclockwise) yields identical results due to the inherent symmetry of pNA, so only one direction is shown, and we report the angles within 0° ≤ φ, θ ≤ 90°.

**Figure 3:**
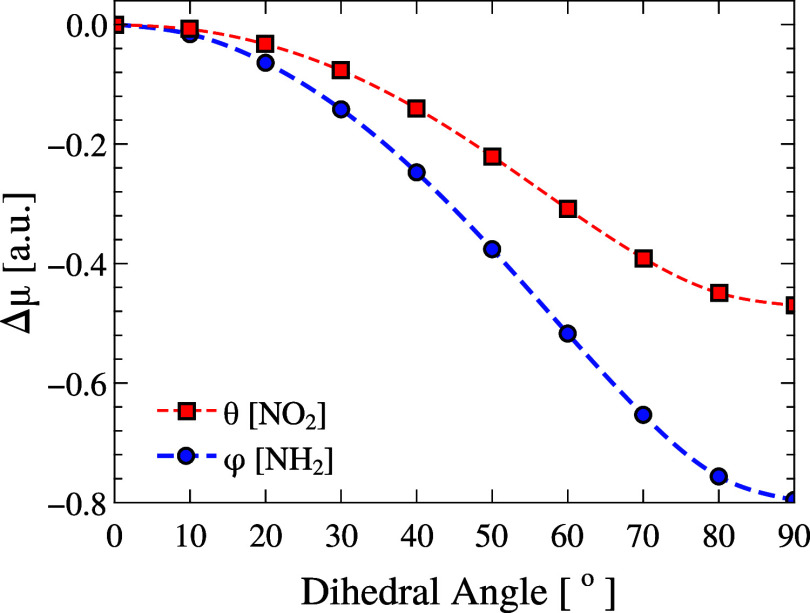
Relative dipole moment (Δμ = μ – μ_gas_, [au]) of pNA as a function of the NH_2_ torsion (φ, blue) and the NO_2_ torsion (θ, red). Both curves are referenced to the equilibrium gas-phase geometry, taken as (φ, θ) = (0°, 0°) with Δμ = 0. NH_2_ rotation induces a dipole reduction nearly twice that of NO_2_, highlighting the dominant donor-side contribution to electronic redistribution.

The equilibrium geometry corresponds to the configuration with the maximum dipole moment. As the rotation angle increases, the rotation of both groups leads to a monotonic decrease in the dipole moment. However, the NH_2_ group induces a more pronounced change, reducing the dipole moment by approximately −0.8 au, compared to less than −0.5 au for the NO_2_ group. This result suggests that the electronic distribution is more sensitive to structural distortions near the donor (NH_2_) moiety than near the acceptor (NO_2_) group. In both cases, deviations from equilibrium produce a decay that qualitatively follows the functional form 1 + cosθ. In particular, the magnitude of these variations exceeds by a factor of 2 the computational uncertainty associated with the level of theory (estimated at 0.26 au), supporting the robustness of both the observed trends and absolute values.

As a compact summary of the torsional dependence, Figure 4 maps the relative dipole, Δμ­(θ, φ) = μ­(θ, φ) – μ­(0, 0), over the dihedrals θ ≡ τ­(OA–N2–C4–C3) (NO_2_ twist) and φ ≡ τ­(HA–N1–C1–C2) (NH_2_ twist). The response is monotonic along both coordinates and markedly steeper along the donor-side rotation (φ). Quantitatively, at fixed θ = 30°, the dipole drops by ∼0.76 au when φ increases from 0° to 90° [μ­(30°, 0°) = 3.00 → μ­(30°, 90°) = 2.24], whereas at fixed φ = 30°, raising θ from 0° to 90° lowers μ by ∼0.44 au [2.94 → 2.49]. The global minimum at (θ, φ) = (90°, 90°) corresponds to Δμ ≈ −1.07 au relative to (0°, 0°) [3.08 → 2.01]. This net decrease is substantially cumulative yet ∼15% smaller in magnitude than the simple sum of the one-coordinate drops [−0.47 – 0.80 = −1.27 au], indicating weak, subadditive coupling between the two torsional coordinates. Consistently, the iso-Δμ contour map (projection of Figure 4 onto the θ–φ plane) shows that for small NH_2_ twists (φ ≲ 30°) the contours are oblique and crowd along θ, revealing a sharp decrease of Δμ with θ at fixed φ (about ∼0.05 au per 10° along φ = 30°), whereas for larger φ they bend toward the diagonal and densify in both directions, consistent with a cooperative reduction culminating near (90°, 90°).

**Figure 4:**
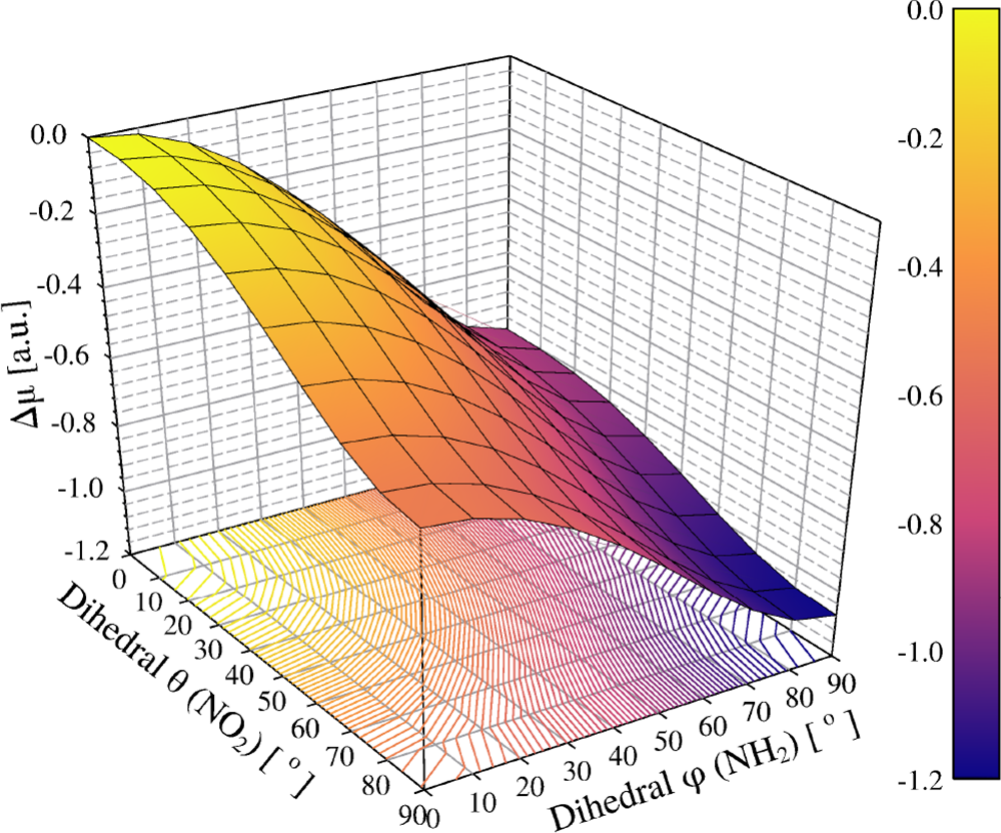
Surface map of the relative dipole, Δμ­(θ, φ) = μ­(θ, φ) – μ­(0, 0) (au), as a function of the dihedral angles θ ≡ τ­(OA–N2–C4–C3) (NO_2_ twist) and φ ≡ τ­(HA–N1–C1–C2) (NH_2_ twist). The response is monotonic along both coordinates and markedly steeper along φ. The global minimum lies near (θ, φ) = (90°, 90°); the overall decrease is largely cumulative yet slightly subadditive, indicating weak coupling between the two torsional coordinates. The reference dipole moment μ­(0, 0) is 3.09 au.

In addition to group rotations, we also investigate the effect of localized bond deformations that mimic vibrational displacements. In this context, the displacements of hydrogen atoms H_2_ and H_3_, which are directly bonded to the aromatic ring and positioned near the donor (NH_2_) and acceptor (NO_2_) moieties, respectively, offer a simplified yet representative model of zero-point motion or mass-induced vibrational changes. Such structural variations can naturally arise in thermally populated vibrational states. The analysis below explores how the dipole moment responds to the symmetric stretching and contraction of these bonds. These perturbations produce dipole moment variations of less than 0.05 au, which are significantly smaller than those induced by group rotations and comparable to the uncertainty associated with the level of theory. This confirms that low-amplitude vibrational effects have only a moderate impact on the electronic distribution. Full analysis, including combination modes and displacement profiles, is presented in the Supporting Information.

In addition to C–H vibrational displacements, we also assessed the impact of deforming a chemically significant bond in the donor region. Figure 5 presents the dipole moment variation relative to its gas-phase equilibrium value as a function of the C_1_–N_1_ bond length. As the C_1_–N_1_ bond is either elongated or shortened relative to its equilibrium value, the dipole moment changes nearly linearly, decreasing with bond elongation and increasing with bond contraction. The total change in dipole moment in the considered range reaches approximately 0.2 au, about five times larger than the variation observed for the combined displacement of the H_3_ and H_6_ hydrogens (Figure S2 in the Supporting Information). Although still slightly smaller than the variation introduced by the level of theory (estimated at 0.26 au), this result highlights the substantial impact that localized bond contractions can exert on the dipole response of the molecule.

**Figure 5:**
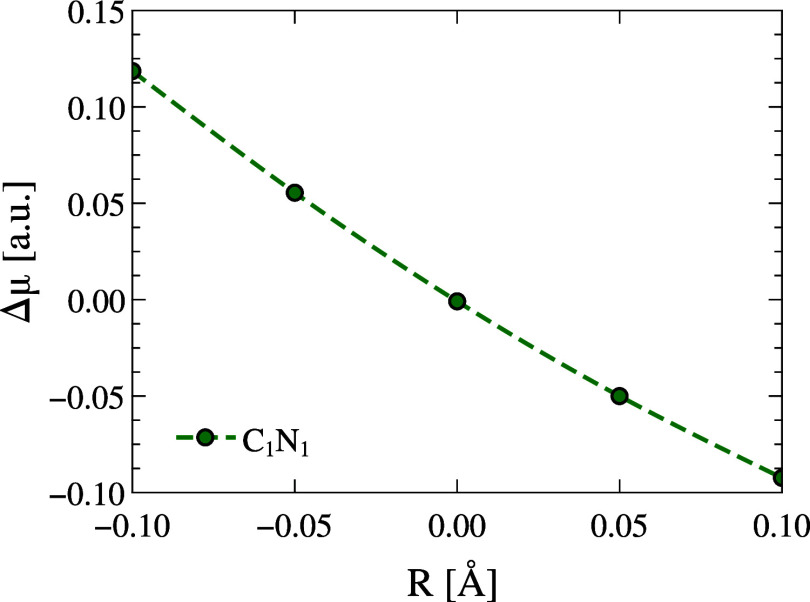
Relative dipole moment (Δμ = μ – μ_gas_, [au]) of pNA as a function of the donor C_1_–N_1_ bond stretching/contracting by ±0.1 Å. The reference point (*R* = 0, Δμ = 0) corresponds to the equilibrium gas-phase geometry, for which the dipole moment is μ_gas_ = 3.087 au. Bond contraction increases the dipole, while elongation decreases it, showing a nearly linear response to localized radial perturbations.

Finally, we examine how the dipole moment responds to the combined effect of the NH_2_ rotation and bond deformation. Figure 6 presents the results for a 90° rotation of the NH_2_ group under two distinct conditions: when the H_3_ bond is stretched (blue squares) or contracted (red circles) by 0.1 Å. Notably, both curves are nearly indistinguishable, indicating that the vibrational perturbation of H_3_ has little to no impact on the overall dipole response induced by NH_2_ rotation. This result aligns with previous observations. As shown in Figure S1 (Supporting Information), the isolated displacement of H_3_ affects the dipole moment by less than 0.015 au, a much smaller variation produced by group rotations. The effect remains negligible even when superimposed on a large amplitude rotational distortion. These results reinforce the conclusion that low-magnitude vibrational motions, such as those arising from hydrogen stretching, contribute only modestly to the electronic redistribution associated with larger structural changes.

**Figure 6:**
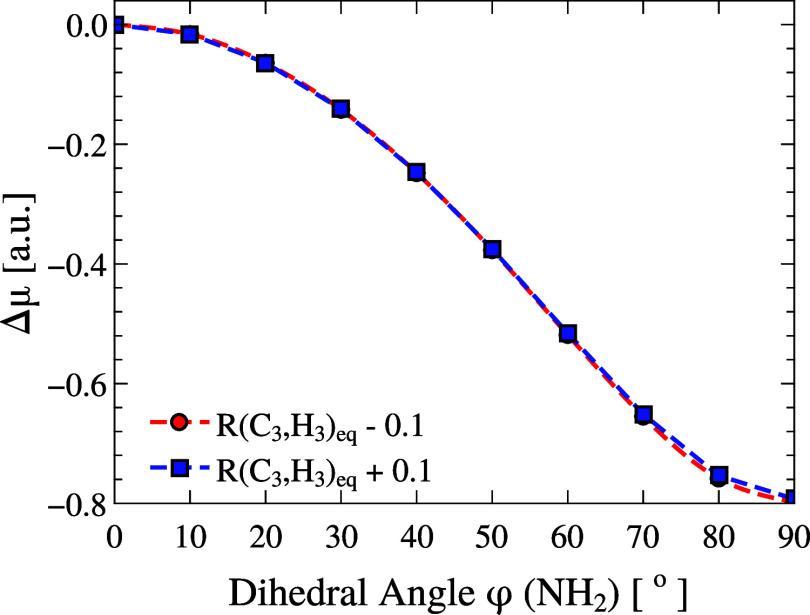
Dipole moment variation during NH_2_ rotation (φ, 0–90°) combined with H3 stretching (blue) or contraction (red) by 0.10 Å around equilibrium. The NO_2_ torsion is kept fixed at θ = 0°. Curves are reported as Δμ = μ – μ­(0, 0) (in au). The steeper slope of the φ scan highlights the dominant donor-side control on μ. The reference point corresponds to the equilibrium gas-phase geometry, for which the dipole moment is μ_gas_ = 3.087 au.

Overall, the dipole moment exhibits two distinct patterns of behavior, depending on the type of structural modification. Rotations of the NH_2_ and NO_2_ groups induce variations that follow a trigonometric-like profile, closely resembling the functional form 1 + cosθ, with clear maxima at the equilibrium geometry. In contrast, bond stretching and contraction, particularly involving hydrogens directly bonded to the aromatic ring, result in nearly linear changes in the dipole moment. Overall, dipole moment variations are dominated by NH_2_ torsion, while NO_2_ rotation has a smaller effect, and local vibrations contribute negligibly. Consistently, the two-dimensional map in Figure 4 consolidates these trends: Δμ decreases monotonically with either dihedral (steeper along the donor-side rotation, φ), reaching a global minimum at large θ and φ. The overall drop at (90°, 90°) is largely cumulative yet ∼15% smaller (in magnitude) than the sum of the single-coordinate changes, indicating weak, subadditive coupling between the two torsional coordinates and reinforcing the robustness of the dipole moment as a structural probe.

### HOMO–LUMO

In addition to their influence on dipole-related properties, structural deformations can also impact the electronic structure of the molecule. In particular, changes in the energies of the highest occupied molecular orbital (HOMO) and lowest unoccupied molecular orbital (LUMO), as well as the corresponding HOMO–LUMO gap, provide helpful insight into how the electronic structure responds to geometric perturbations. These variations are especially relevant in systems like pNA, where donor and acceptor groups are conjugated through a π-system, and frontier orbitals are expected to be highly sensitive to torsional and electronic effects.

Figure 7 shows the variation in HOMO and LUMO energies, as well as in the HOMO–LUMO gap, as a function of a 90° rotation of the NH_2_ group (left) and the NO_2_ group (right) around the main molecular axis. All of the values are presented relative to their corresponding gas-phase equilibrium values.

**Figure 7:**
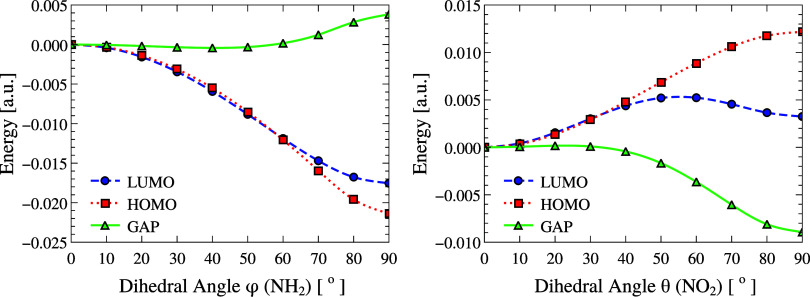
HOMO (red), LUMO (blue), and HOMO–LUMO gap (green) energies of pNA as a function of the donor torsion φ (NH_2_, left) and acceptor torsion θ (NO_2_, right). Energies are reported relative to the equilibrium gas-phase geometry, taken as (φ, θ) = (0°, 0°) with Δ*E* = 0. NH_2_ rotation preserves the gap up to ∼60°, whereas NO_2_ rotation steadily decreases it, reflecting donor–acceptor asymmetry. The reference HOMO and LUMO energies at equilibrium are −0.2371 and −0.0857 au, respectively, resulting in a HOMO–LUMO gap of 0.1514 au.

The rotational patterns of the two groups differ markedly. For NH_2_, HOMO and LUMO energies decrease almost in parallel up to ∼60°, keeping the HOMO–LUMO gap nearly constant. Beyond this threshold, HOMO decreases more steeply, producing a noticeable gap increase. In contrast, NO_2_ rotation shows the opposite trend: both frontier orbital energies increase, and the gap decreases steadily, reflecting the distinct donor–acceptor roles and the electronic asymmetry already indicated by the dipole moment response (Section “Dipole Moment”).

The impact of the computational level is significantly larger, with differences of 0.138 au for LUMO, 0.087 au for HOMO, and 0.224 au for the HOMO–LUMO gap (Table 1), at least 1 order of magnitude greater than those induced by rotations.

Figure 8 summarizes the joint effect of the two torsions: the shaded gray surface is the HOMO–LUMO gap Δ*E*, while the red/blue surfaces are *E*_HOMO_/*E*_LUMO_ (all referenced to (0°, 0°)). For a small NO_2_ twist (θ ≈ 0°), the gap is only weakly sensitive to the NH_2_ rotation: increasing φ from 0° to 90° widens Δ*E* by just +0.0038 au (0.15154 → 0.15537). In contrast, for a large NO_2_ twist (θ ≈ 90°), the φ-response becomes strong, with a +0.0220 au increase (0.14261 → 0.16459), revealing that the effect of φ is amplified at high θ. Along θ, the behavior reverses with φ: at φ = 0°, the gap narrows by −0.0089 au (0.15154 → 0.14261), whereas at φ = 90°, it widens by +0.0092 au (0.15537 → 0.16459). The wireframes clarify this crossover: along φ, both frontier levels are stabilized (shift downward) with a larger magnitude for the HOMO, while along θ, both are destabilized (shift upward) and the relative rates swap with φ, yielding the observed change in the θ-trend of Δ*E*. Overall, the gap is smooth across the plane, minimal at (θ, φ) = (90°, 0°) and maximal at (90°, 90°).

**Figure 8:**
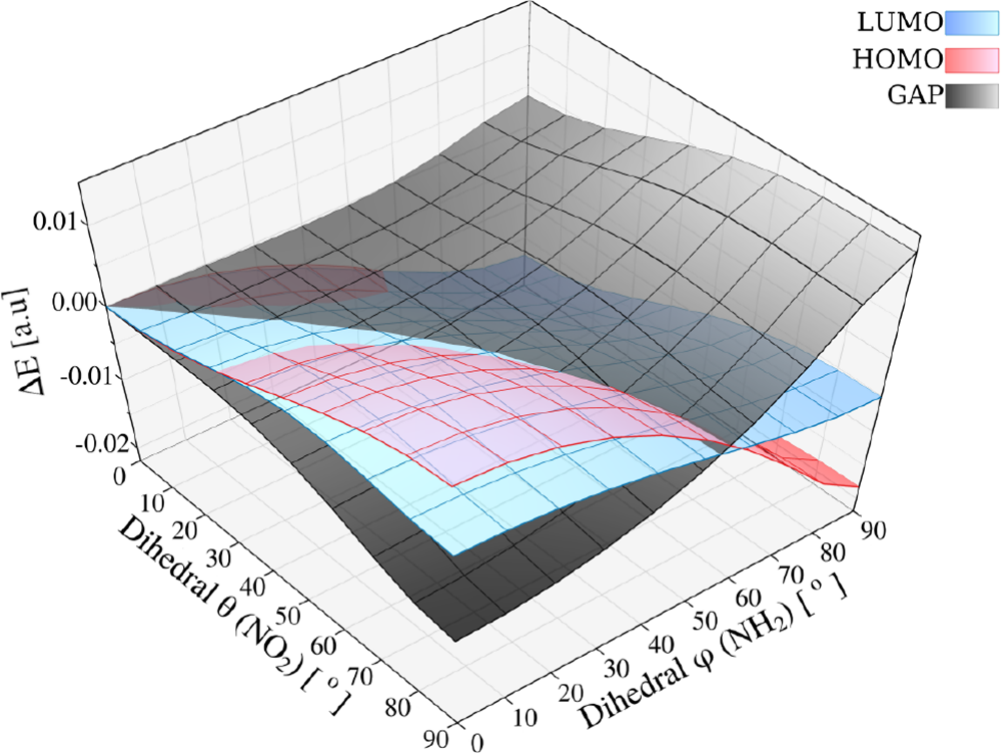
Relative frontier-orbital energies of pNA (Δ*E*_*X*_ = *X*(φ, θ) – *X*(0°, 0°) for *X* ∈ {*E*_HOMO_, *E*_LUMO_, and *E*_gap_}) as a function of the NH_2_ torsion φ and NO_2_ torsion θ. The filled gray surface shows Δ*E*_gap_, while the red and blue wireframes and surfaces show Δ*E*_HOMO_ and Δ*E*_LUMO_, respectively. The reference HOMO and LUMO energies at equilibrium are −0.2371 and −0.0857 au, respectively, resulting in a HOMO–LUMO gap of 0.1514 au.

Localized vibrational displacements, such as H_2_ and H_3_ stretching, produce variations below 10^–3^ au and do not significantly alter the gap, while the deformation of the C_1_–N_1_ bond induces only moderate shifts (<10^–2^ au, about one-tenth of the effect of the computational level). Complete orbital profiles and quantitative analyses are provided in the Supporting Information.

In addition to combined hydrogen displacements, we analyzed the deformation of the C_1_–N_1_ bond and the coupling between the torsional and vibrational motions. These local distortions produce only minor variations in HOMO, LUMO, and the HOMO–LUMO gap (<0.03 au), roughly one-tenth of the changes caused by the choice of the computational level. Even when NH_2_ rotation is combined with H_3_ stretching or contraction, the total orbital variation remains small, confirming that localized vibrational effects have a negligible impact on the electronic structure compared to group rotations and methodological differences.

In summary, torsional motions dominate the frontier–orbital response. The NH_2_ rotation (φ) is the main driver of the HOMO–LUMO gap: its effect is modest near θ ≈ 0° but becomes pronounced at large NO_2_ twists, leading to a clear widening of Δ*E*. The NO_2_ rotation (θ) provides a secondary, φ-dependent modulation, reducing the gap when φ ≈ 0° and increasing it when φ ≈ 90°. Localized vibrations contribute negligibly, indicating that the electronic structure is governed by cooperative torsional tuning with weak, subadditive coupling.

### Polarizability α

Figure 9 shows the variation in the polarizability α resulting from a 90° rotation of the NH_2_ group (blue circles) and the NO_2_ group (red squares) around the main molecular axis. The calculated values are presented relative to the gas-phase equilibrium reference given in Table 1.

**Figure 9:**
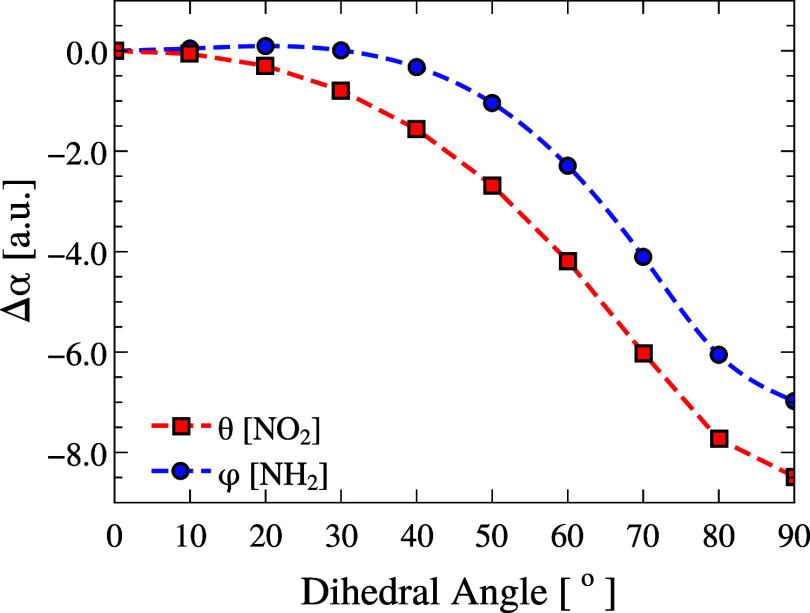
Relative isotropic polarizability (Δα = α – α_gas_, [au]) of pNA as a function of the donor torsion φ (NH_2_, blue) and the acceptor torsion θ (NO_2_, red). The reference point (φ, θ) = (0°, 0°) corresponds to the equilibrium gas-phase geometry with Δα = 0. NO_2_ rotation causes the largest variation (∼8.5 au), whereas NH_2_ rotation shows a threshold-like response, remaining stable up to ∼30°. The reference isotropic polarizability at the equilibrium geometry is α_gas_ = 104.3 au.

The rotation of both groups results in curves with similar shapes but different magnitudes. The NO_2_ group causes the largest change (∼8.5 au), whereas for NH_2_, the polarizability remains nearly constant up to ∼30°, indicating a stable structural regime. This behavior confirms the presence of a threshold angle: ∼30° for NH_2_ and below 20° for NO_2_. Below these values, small rotations do not significantly change α, suggesting that the refractive index remains stable under mild torsional changes. Compared to the variation introduced by changing the level of theory (∼4.3 au), the effect of group rotation on α is about twice as large, highlighting its strong sensitivity to torsional deformations relative to methodological choices.

Figure 10 shows the polarizability surface (au) as a function of the dihedral angles θ (NO_2_ twist) and φ (NH_2_ twist). The polarizability surface decreases monotonically with both dihedral angles, and the response of one torsion is amplified when the other is small and saturates when the other is large. Quantitatively, at fixed φ = 0°, the NO_2_ rotation (θ) reduces the polarizability by −8.48 au as θ goes from 0° to 90° [104.810 → 96.327], whereas at φ = 90°, the same scan in θ has a weaker effect, −3.43 au [97.832 → 94.400]. Conversely, the NH_2_ rotation (φ) is strong when θ = 0° (−6.98 au, 104.810 → 97.832) but much smaller at θ = 90° (−1.93 au, 96.327 → 94.400). This complementary behavior produces a steep ridge near small angles and a progressive flattening toward large angles, with the minimum at (θ, φ) ≈ (90°, 90°) corresponding to a net change of Δθ ≈ −10.41 au relative to (0°, 0°).

**Figure 10:**
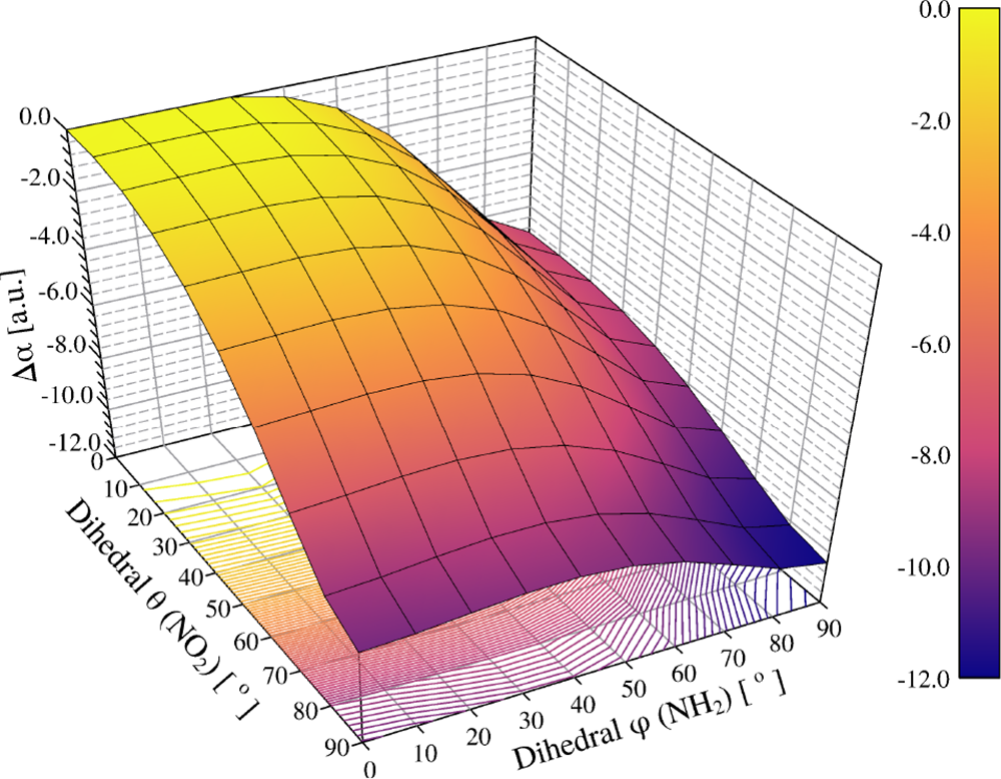
Polarizability surface (au) as a function of the dihedral angles θ (NO_2_ twist) and φ (NH_2_ twist). Values are shown relative to (θ, φ) = (0°, 0°). The surface decreases monotonically with either rotation; the sensitivity is strong when the other torsion is small [e.g., at φ = 0°: Δθ(0° → 90°) → −8.48 au; at θ = 0°: Δφ(0° → 90°) → −6.98 au] and saturates for large orthogonal torsion [at φ = 90°: −3.43 au; at θ = 90°: −1.93 au]. The minimum lies near (90°, 90°), with an overall change of ≈ −10.41 au. The reference isotropic polarizability at the equilibrium geometry is α_gas_ = 104.3 au.

Localized vibrational and bond deformations, such as H2/H3 stretching and C1–N1 variation, produce quasi-linear changes in α with total variations below 3 au, less than half the torsional effect. Even in combined torsional–vibrational scenarios, the impact remains modest. In brief, the molecular polarizability α is mainly governed by the NO_2_ rotation, whereas its response to the NH_2_ twist is threshold-like and is essentially flat for φ ≲ 30° and decreasing only beyond that. Local bond stretches and contractions add only small quasilinear offsets, confirming that torsional degrees of freedom dominate the polarizability response.

### Hyperpolarizability β

Following the analysis of α, we now examine how structural deformations affect the first hyperpolarizability β. Figure 11 shows its variation relative to the gas-phase equilibrium value (Table 1) under a 90° rotation of the NH_2_ group (blue circles) and the NO_2_ group (red squares).

**Figure 11:**
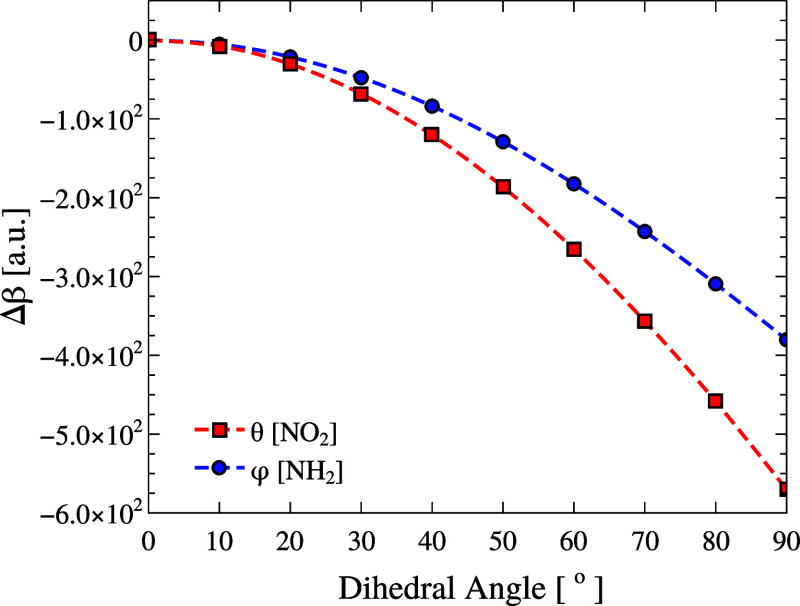
Relative first hyperpolarizability (Δβ = β – β_gas_, [au]) of pNA as a function of the donor torsion φ (NH_2_, blue) and the acceptor torsion θ (NO_2_, red). The reference point (φ, θ) = (0°, 0°) corresponds to the equilibrium gas-phase geometry with Δβ_HRS_ = 0. The reference value of the total hyper-Rayleigh scattering first hyperpolarizability at equilibrium is β_HRS,gas_ = 1.37 × 10^3^ au.

For NH_2_, both dipolar and octupolar contributions remain nearly constant up to 50°, followed by a slight increase and a pronounced decrease of ∼3.8 × 10^2^ au, corresponding to about a 28% reduction in β_HRS_ as the torsion approaches 90°. In contrast, NO_2_ rotation yields a similar overall pattern, but the amplitude of the change is slightly larger, reaching ∼5.7 × 10^2^ au, or about 42% relative to the equilibrium value, reflecting a stronger sensitivity of the acceptor torsion. In both cases, a threshold angle is evident, indicating that small-amplitude oscillations around the equilibrium geometry have a minor effect on β_HRS_, consistent with the smooth torsional dependence observed.

As shown in Table 1, changing the level of theory leads to variations of about 5 × 10^2^–8 × 10^2^ au in β_HRS_, corresponding to roughly 40–60% of the gas-phase reference value. These differences are comparable to those induced by torsional rotation, indicating that both methodological and geometric factors play significant roles in determining the hyperpolarizability. Nevertheless, the smooth trend observed along the torsional coordinate suggests that the geometrical dependence is more systematic, reflecting the intrinsic coupling between the planarity and charge-transfer efficiency.

Figure 12 shows the relative HRS first hyperpolarizability (Δβ) as a function of the donor and acceptor torsions. The surface is approximately symmetric with respect to the exchange of φ (NH_2_) and θ (NO_2_), indicating that both rotations contribute comparably to the loss of planarity and conjugation. Increasing either dihedral angle causes a monotonic decrease in β, which approaches its minimum value as the molecule becomes fully twisted. When one group is already highly rotated (near 90°), additional torsion of the other produces only a minor change, confirming that suppression of the nonlinear response arises mainly from the overall structural deplanarization rather than from the specific site of rotation.

**Figure 12:**
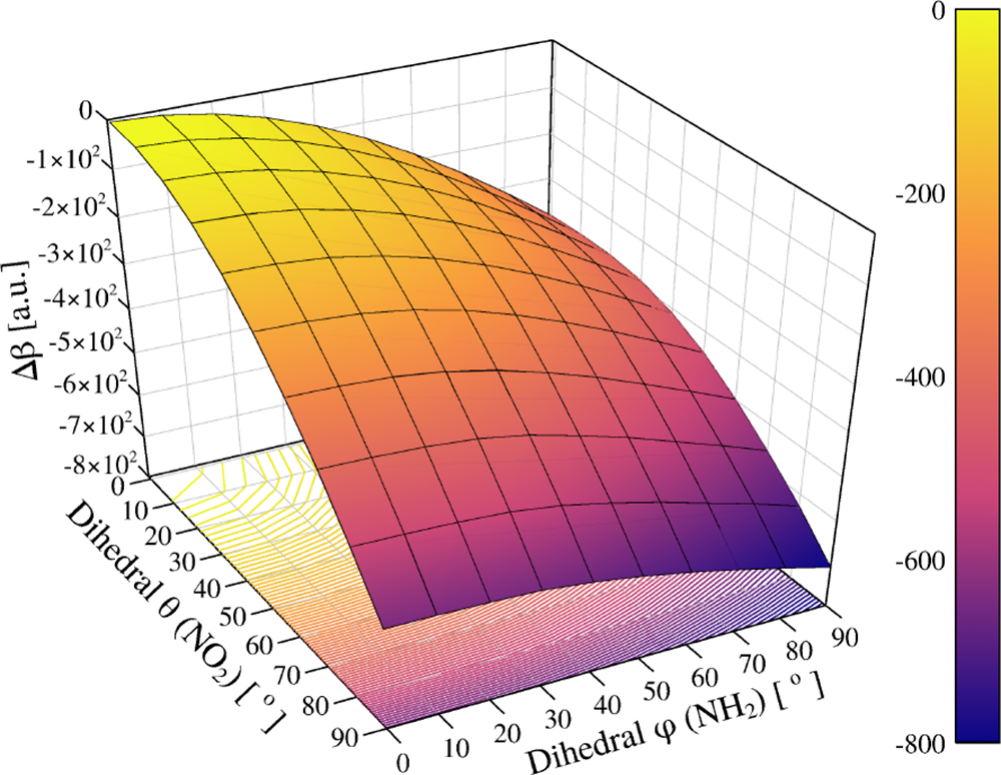
Relative hyper-Rayleigh scattering first hyperpolarizability (Δβ = β_HRS_ – β_HRS,gas_, [au]) of pNA as a function of the dihedrals φ (NH_2_) and θ (NO_2_). The reference (φ, θ) = (0°, 0°) corresponds to the equilibrium gas-phase geometry with Δβ = 0. Both torsions lead to a smooth, correlated decrease in β_HRS_, primarily governed by the overall loss of planarity and conjugation. At equilibrium, the total hyper-Rayleigh scattering first hyperpolarizability is β = 1.37 × 10^3^ au, which serves as the reference for all relative values.

Localized and coupled vibrational effects, including H2/H3 stretching, C1–N1 bond variation, and torsion–vibration combinations, introduce only minor additional changes. Overall, β decreases smoothly with torsion, showing a gradual loss of nonlinear response as either dihedral angle increases. The reduction is monotonic and primarily associated with the progressive loss of planarity and conjugation, while vibrational couplings produce only minor additional effects.

### Hyperpolarizability γ

Figure 13 shows the effect of 90° rotations of the NH_2_ (blue circles) and NO_2_ (red squares) groups on the second hyperpolarizability γ, relative to its gas-phase equilibrium value (Table 1). In both cases, a distinct resonance-like feature appears: around 75° for NH_2_ and near 35° for NO_2_. Outside these angles, γ remains essentially unchanged with values close to zero throughout the rest of the torsional range. These apparent resonances do not correspond to real optical excitations but rather to near-resonant electronic contributions within the two-state charge-transfer model. The torsional deformations modulate the donor–acceptor energy gap, transiently enhancing the dynamic third-order response at the applied frequency (0.043 au), consistent with the observed peaks.

**Figure 13:**
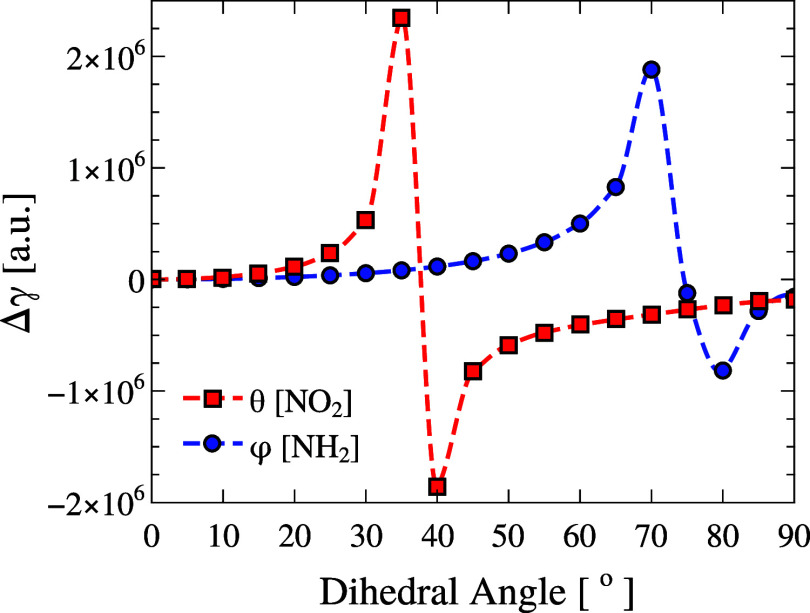
Relative second hyperpolarizability (Δγ = γ – γ_gas_, [au]) of pNA as a function of the donor torsion φ (NH_2_, blue) and the acceptor torsion θ (NO_2_, red). The reference point (φ, θ) = (0°, 0°) corresponds to the equilibrium gas-phase geometry with Δγ = 0. γ remains nearly constant across most angles, with resonant-like peaks at φ ≈ 75° (NH_2_) and θ ≈ 35° (NO_2_), confirming that large-amplitude torsions dominate third-order responses. The reference second hyperpolarizability at the equilibrium geometry is γ_gas_ = 2.16 × 10^5^ au.

This behavior reflects the same trend observed for α and β: torsional deformations dominate the response, while localized vibrational and bond distortions introduce only minor, quasi-linear variations. Even when torsional and vibrational modes are combined, the main resonant-like features and their positions are preserved, confirming that γ is largely controlled by large-amplitude rotations.

Figure 14 demonstrates that the second hyperpolarizability of *p*-nitroaniline is the most sensitive property to structural distortions among those investigated. Unlike the dipole, α, or even β, the γ response exhibits sharp resonance-like features when the NH_2_ and NO_2_ groups are simultaneously rotated away from planarity. These peaks arise from the disruption of π-conjugation and charge-transfer interactions, which strongly modulate the third-order response. The observed resonances at φ ≈ 75° (NH_2_) and θ ≈ 35° (NO_2_) confirm the nonlinear and cooperative character of the torsional effects. Experimentally, such behavior could manifest as enhanced or quenched signals in third-harmonic generation (THG) or nonlinear refractive index measurements, reinforcing that maintaining donor–acceptor coplanarity is crucial for optimizing third-order NLO performance.

**Figure 14:**
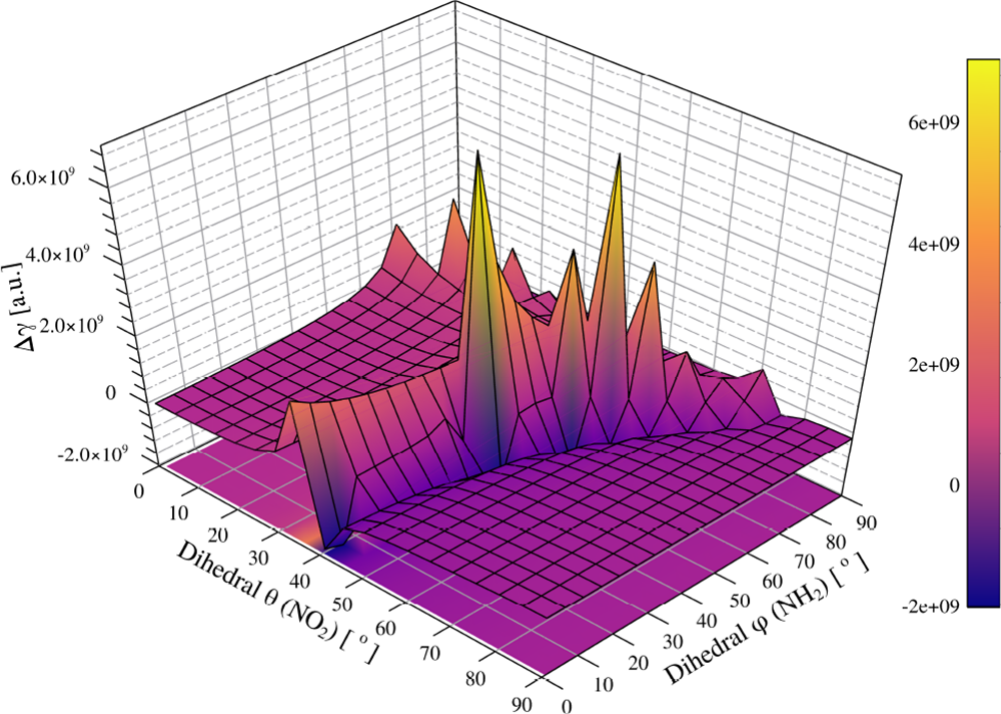
Relative second hyperpolarizability (Δγ = γ – γ_gas_, [au]) of pNA as a function of the dihedrals φ (NH_2_) and θ (NO_2_). The equilibrium gas-phase geometry (φ, θ) = (0°, 0°) is taken as a reference with Δγ = 0. The response surface shows pronounced variations, including resonance-like peaks at specific torsional combinations, indicating the strong sensitivity of γ to deviations from planarity. The reference second hyperpolarizability at the equilibrium geometry is γ_gas_ = 2.16 × 10^5^ au.

In summary, γ remains nearly invariant under small distortions, with only large-amplitude torsions producing resonant-like peaks (75° for NH_2_ and 35° for NO_2_). This confirms that third-order responses are predominantly governed by major torsional motions, with a negligible contribution from local vibrations.

### Interpretation of Structural Effects

In extended π-conjugated systems, variations in conjugation and NLO responses are often governed by bond-length alternation (BLA), which modulates electronic delocalization and the response to the solvent environment and/or external fields.^2,52,53^ In contrast, for *p*-nitroaniline, the π-conjugated aromatic ring behaves as an almost rigid unit, exhibiting negligible bond-length variation compared to the torsional and donor–acceptor distortions explored here. This intrinsic rigidity of the aromatic core defines the structural baseline upon which the deformations considered here act. Consequently, BLA effects are not explicitly discussed in the present work. For comprehensive analyses connecting geometric and polarization effects, see refs 2, 52 and 53 .

The influence of structural modifications on NLO properties is clear: torsions of the donor (NH_2_) and acceptor (NO_2_) groups directly modulate the conjugation pathway and the underlying charge transfer along the D−π–A system. Although the absolute values of α, β, and γ may differ with the computational method, the qualitative trends linked to these distortions remain consistent and provide the central basis for interpreting the geometry–property relationships discussed in this work. These considerations do not diminish the relevance of the calculated values; rather, they highlight the need to interpret the results within the appropriate theoretical framework and in light of experimental observables, such as HRS or second-harmonic generation, which are sensitive to similar geometric distortions.

The calculated β value for *p*-nitroaniline in the gas phase is 1.37 × 10^3^ au (1.2 × 10^–29^ esu). This result is consistent with the theoretical range reported by Kaka et al.^54^ (5.2 – 8.1 × 10^2^ au) and also agrees remarkably well with the experimental data of Woodford et al.^55^ In that work, the authors measured β for pNA in CHCl_3_ and DMSO and subsequently removed the solvent contribution by using local-field and dispersion corrections based on the Onsager model. The resulting intrinsic values, β_0_ = (1.18 ± 0.15) × 10^–29^ and (1.15 ± 0.15) × 10^–29^ esu, respectively, are in very good agreement with our gas-phase result. This close agreement confirms that the computed response captures the intrinsic electronic contribution to the NLO behavior, while solvent effects primarily rescale the overall magnitude.

The calculated third-order hyperpolarizability for *p*-nitroaniline in the gas phase (γ = 2.16 × 10^5^ au) corresponds to the total dynamic response at finite frequency. In contrast, Kaka et al.^54^ reported static values of γ_∥_ = 3.8 × 10^4^ and γ_THS_ = 5.5 × 10^4^ au at the CCSD­(T) level. Since their analysis indicated that γ­(ω) increases with frequency up to near-resonant conditions, the present value, obtained at *ℏ*ω = 1.17 eV, is expected to be higher. Therefore, although the numerical values are not directly comparable due to the different frequency regimes and tensor definitions, they exhibit the same order of magnitude and reflect the consistent enhancement of γ under optical excitation.

As observed in the previous sections, the amplitude of response follows the hierarchy: dipole < α < β < γ, with torsional deformations of NH_2_ and NO_2_ being the dominant factor. Localized hydrogen or C1 and N1 displacements introduce only minor quasi-linear variations, and torsion–vibration combinations do not qualitatively alter the main response surfaces. This confirms that the key structure–property relationships are governed by large-amplitude rotations.

Although the present investigation is fully theoretical, its findings have relevant experimental implications. The structural deformations modeled here, such as group rotations and localized bond distortions, mimic realistic perturbations that arise under experimental conditions, including thermal fluctuations, vibrational dynamics, and structural disorder in condensed phases. In such environments, molecules seldom remain at their equilibrium geometry; instead, they populate a distribution of conformations that can significantly influence their optical behavior. Several experimental studies involving pNA in solution or crystalline phases have demonstrated the sensitivity of NLO properties to such conformational changes and the solvent environment,^23–27,33–39^ particularly in the context of HRS and second harmonic generation (SHG) measurements. The present results help clarify the microscopic origin of these effects by isolating how specific structural degrees of freedom affect dipole moments and (hyper)­polarizabilities. Moreover, the computational framework adopted here can be readily extended to other push–pull chromophores, where similar structure–property relationships are expected. These insights contribute to the rational design of NLO-active molecules with tunable responses, especially in functional environments, such as solvents or polymers.

## Thermal Ensemble Modeling: Bridging Structural Fluctuations and NLO Properties

After analyzing the effects of individual structural deformations on the dipole moment, polarizabilities, and hyperpolarizabilities, we now consider how these distortions contribute statistically to the behavior of an ensemble of molecules at a finite temperature.

Molecular configurations resulting from different degrees of rotation, contraction, or stretching possess distinct total free energies *E*. In a thermally populated ensemble, the probability of each configuration *i* relative to the ground-state geometry is given by the Boltzmann expression: *p*_*i*_ = exp­[−(*E*_*i*_ – *E*_0_)/*KT*], where *E*_0_ is the free energy of the equilibrium configuration. As illustrated in Table 2, and consistent with the results discussed earlier, the planar configuration of the NH_2_ group (i.e., without rotation, see Figure 1) corresponds to the minimum energy structure. Similar calculations were performed for the rotation of the NO_2_ group as well as for the stretching and contraction of the H2 and H3 bonds, including combined deformations with H6. These structural variations provide a thermal ensemble of geometries whose relative populations can now be evaluated in the context of their energetic accessibility.

**Table 2:** Free Energy and Probability at *T* = 300 K for NH_2_ Bond Rotation

angle (°)	*E* (10^–15^ J)	probability
0	–2.14574	1.00
10	–2.14574	5.84 × 10^–1^
20	–2.14573	1.26 × 10^–1^
30	–2.14572	1.2 × 10^–2^
40	–2.14571	6.95 × 10^–4^
50	–2.14569	2.13 × 10^–5^
60	–2.14568	5.97 × 10^–7^
70	–2.14566	2.42 × 10^–9^
80	–2.14566	4.3 × 10^–9^
90	–2.14566	2.42 × 10^–9^

The probability associated with each configuration allows us to compute the thermally averaged NLO properties across the ensemble. For instance, at finite temperature (*T* ≠ 0), the polarizability is given by the weighted expression α = ∑_*i*_ *p*_*i*_α_*i*_/∑_*i*_ *p*_*i*_, where α_*i*_ is the polarizability calculated for configuration *i* using the DALTON program.^42^

Following this procedure, we can determine the thermal averages of all properties at various temperatures, as summarized in Table 3. The selected temperatures—298 K (room temperature), 373 K (boiling water), 421 K (pNA fusion), and 605 K (pNA boiling)—span a range of practical physical conditions. Although the differences appear small, even low-probability conformations can contribute to optical responses in condensed phases, where environmental interactions and vibrational coupling enhance these effects. As shown in the table, the thermally averaged values differ only slightly from those obtained for the equilibrium configuration, indicating a limited thermal sensitivity under the modeled distortions.

**Table 3:** Ensemble NLO Properties at Various Temperatures^a^

*T* (K)	DM^b^	α (×10^2^)	β^*J*=1^ (×10^6^)	β^*J*=3^ (×10^6^)	γ (×10^5^)
298	3071	1049	5807	6165	2162
373	3070	1049	5806	6161	2166
421	3069	1049	5804	6158	2169
605	3064	1049	5797	6146	2166

a All quantities are given in atomic units (au).

b The calculations were performed at the Hartree–Fock level.

To summarize, the ensemble analysis confirms that the NLO properties of pNA are remarkably robust under thermal fluctuations. Despite the structural distortions sampled across the thermally populated configurations, the ensemble-averaged values of the dipole moment, polarizability, and hyperpolarizabilities vary only slightly over a broad temperature range. This result reinforces the reliability of single-geometry predictions for pNA and highlights the stability of its NLO response, even under thermally induced structural disorder.

## Conclusions

The effects of bond stretching/contraction and group rotations on the linear and NLO properties of *p*-nitroaniline (pNA) were analyzed by using a systematic computational approach. These structural variations mimic internal vibrational dynamics and environmental effects, such as isotopic substitution or thermal excitation, allowing a direct assessment of their influence on NLO responses. Rotations of NH_2_ and NO_2_, as well as bond deformations, tend to reduce the dipole moment with a quasi-linear response to stretching. Rotational effects on the electronic structure differ between groups: NH_2_ rotation lowers both HOMO and LUMO energies while preserving the gap up to 50°, whereas NO_2_ rotation leads to a simultaneous increase in both orbitals up to 40°. Stretching bonds near NH_2_ increases the LUMO energy, while deformation of bonds associated with NO_2_ decreases it. Polarizability (α) remains stable up to 30° of NH_2_ rotation and then decreases monotonically, a behavior unaffected by additional H3 bond deformations. Stretching hydrogens near the main structure increases α, while elongating the NH_2_-linking bond reduces it. The first hyperpolarizability (β) shows a threshold-dependent variation. Rotations beyond 40–50° induce abrupt changes, especially in the octupolar contribution. Stretching the NH_2_ group or its adjacent bonds causes a splitting between β_*J*=1_ and β_*J*=3_, revealing sensitivity to geometric asymmetry and electronic delocalization, characteristics favorable for tunable NLO behavior. The second hyperpolarizability (γ) remains nearly unchanged throughout most deformations, with particular resonances occurring at 75° for NH_2_ and 35° for NO_2_. While γ increases with bond stretching near NH_2_, it decreases with similar deformations near NO_2_. Although its variation is more limited than that of β, γ still encodes relevant geometric dependencies for third-order NLO responses. Overall, the results confirm that purely geometric distortions, even in the absence of solvent or external field effects, govern the intrinsic hierarchy of NLO responses in pNA, revealing clear threshold-like behaviors that link molecular geometry to optical properties. These findings highlight which structural degrees of freedom dominate the response and which deformations leave it robust, providing a concise physical picture of geometry-driven NLO modulation.

Importantly, the computed β and γ values are in very good agreement with both theoretical and experimental data reported in the literature, reproducing the same order of magnitude and relative trends under comparable conditions. This consistency reinforces the reliability of this approach for describing the intrinsic electronic contributions to NLO behavior.

By bridging static deformations with ensemble-averaged thermal modeling, this work establishes the intrinsic robustness of pNA’s optical properties under thermally accessible distortions. These insights provide a rational basis for designing push–pull chromophores with enhanced and tunable optical responses while supporting pNA as a benchmark system for fundamental structure–property studies. They also encourage future experimental studies combining NLO measurements with vibrational spectroscopy to clarify how structural dynamics, particularly under thermal or environmental fluctuations, affect the intrinsic NLO behavior in molecular materials.

## Supplementary Material



## Notes

NBO charge analysis at different torsional geometries, the effects of localized bond and vibrational displacements on dipole moment, orbital energies, polarizabilities, and hyperpolarizabilities, as well as combined torsion–vibration coupling, and a complete set of computed IR frequencies and intensities (PDF)
