# Supplementary material for: Geometry-Driven Control of Linear and Nonlinear Optical Responses in p‑Nitroaniline: Insights into Structure–Property Relationships and Thermal Robustness
Source: ACS Omega. 2025 Dec 4;10(49):60477–90. doi: 10.1021/acsomega.5c08003 (PMC12713450; doi:10.1021/acsomega.5c08003)
Supplement: Supplementary file 1 [file ao5c08003_si_001.pdf]

Geometry-Driven Control of Linear and Nonlinear Optical Responses in p-Nitroaniline: Insights into Structure-Property Relationships and Thermal Robustness

Vinicius Manzoni[a], Rodrigo M. Gester[b], Antônio R. da Cunha[c], Gabriel I. Pagola[d], Guillermo F. Quinteiro Rosen[e], Patricio F. Provasi[f]

[a]*Instituto de Física, Universidade Federal de Alagoas, 57072-970, Maceió-AL, Brazil*
[b]*Faculdade de Física, Universidade Federal do Sul e Sudeste do Pará, Marabá, PA 68507-590, Brazil*
[c]*Universidade Federal do Maranhão, UFMA, Campus Balsas, CEP 65800-000, Maranhão, Brazil*
[d]*Universidad de Buenos Aires, Facultad de Ciencias Exactas y Naturales, Departamento de Física, and CONICET–Universidad de Buenos Aires, Instituto de Física de Buenos Aires (IFIBA), Ciudad Universitaria, 1428 Buenos Aires, Argentina.*
[e]*Departamento de Física, Facultad de Ciencias Exactas y Naturales y Agrimensura, Universidad Nacional del Nordeste, IMIT-CONICET, Av. Libertad 5470 Corrientes, Argentina.*
[f]*Department of Physics - University of Northeastern, IMIT-CONICET, Av. Libertad 5500, Corrientes, Argentina.*

SUPPORTING INFORMATION

This document provides the detailed analysis of localized vibrational displacements and bond deformations in *p*-nitroaniline (pNA), complementing the main manuscript. The results focus on the effects of small-amplitude perturbations on the dipole moment, frontier orbital energies, and nonlinear optical (NLO) properties.

Table S1: NBO charges (in e) of key donor and acceptor atoms in p-nitroaniline at different torsional geometries calculated at B3LYP/6-311++G** level. The data show that the nitro oxygens are more negative in the planar geometry and become less negative upon torsion, indicating a reduction of the ground-state push–pull character.

Geometry (φ, θ)	$q(N_{NH2})$	$q(O_{12})$	$q(O_{13})$	$q(N_{NO2})$
Planar (0°, 0°)	–0.905	–0.399	–0.399	+0.481
NH_2 twisted (90°, 0°)	–0.902	–0.382	–0.382	+0.481
NO_2 twisted (0°, 90°)	–0.902	–0.357	–0.357	+0.478
Both twisted (90°, 90°)	–0.902	–0.351	–0.351	+0.475

Effect of Localized Displacements on the Dipole Moment

Figure S1 shows the results for the symmetric stretching and contraction of the H_3 (blue circles) and H_2 (red squares) bonds, simulating vibrational motion or isotope substitution. The dipole moment variations are small (≤ 0.02 a.u.), linear with displacement, and negligible compared to group rotations.

The combined stretching of H_3 and H_6 was also evaluated (Figure S2). The maximum variation is ≈ 0.045 a.u., only 5–6% of the difference introduced by the choice of computational method, indicating its secondary contribution.

Email address: vmanzoni@fis.ufal.br (Vinicius Manzoni)

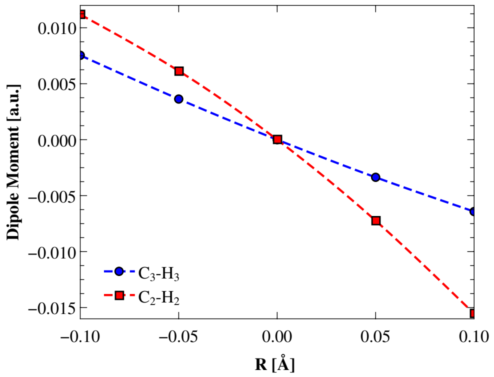

Figure S1: Dipole moment, relative to its gas-phase equilibrium value [in a.u.], of the pNA molecule as a function of bond stretching/contraction by 0.1 Å for H_3 (blue circles) and H_2 (red squares).

Figure S2: Dipole moment, relative to its gas-phase value ($\mu - \mu_{gas}$) [a.u.] of pNA as a function of combined stretching/contraction (±0.1 Å) of H_3 and H_6.

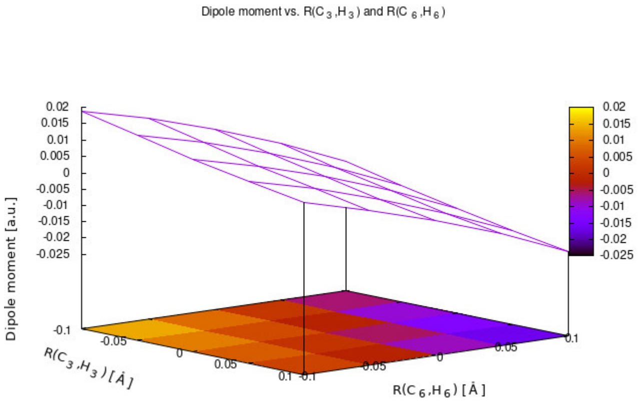

Effect of Localized Displacements on Frontier Orbital Energies

Localized displacements produce much smaller orbital shifts than group rotations. Figure S3 presents the variations in HOMO, LUMO, and HOMO–LUMO gap for symmetric stretching/contraction of H_3 and H_2.

Figure S4 shows the combined effect of stretching H_3 and H_6. The variations remain below 10^{-3} a.u., indicating negligible impact on the electronic structure.

Figure S3: HOMO–LUMO and gap energies, relative to their gas-phase equilibrium value [a.u.], of pNA as a function of stretching/contraction by 0.1 Å for (left) H_3 and (right) H_2.

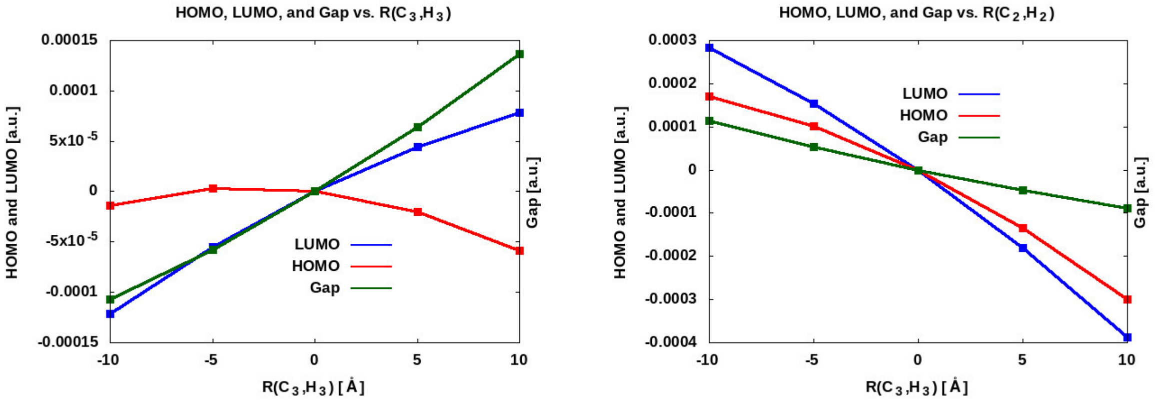

Figure S4: LUMO (left), HOMO (center), and gap (right) energies, relative to gas-phase equilibrium [a.u.], of pNA as a function of combined H_3/H_6 stretching/contraction (±0.1 Å).

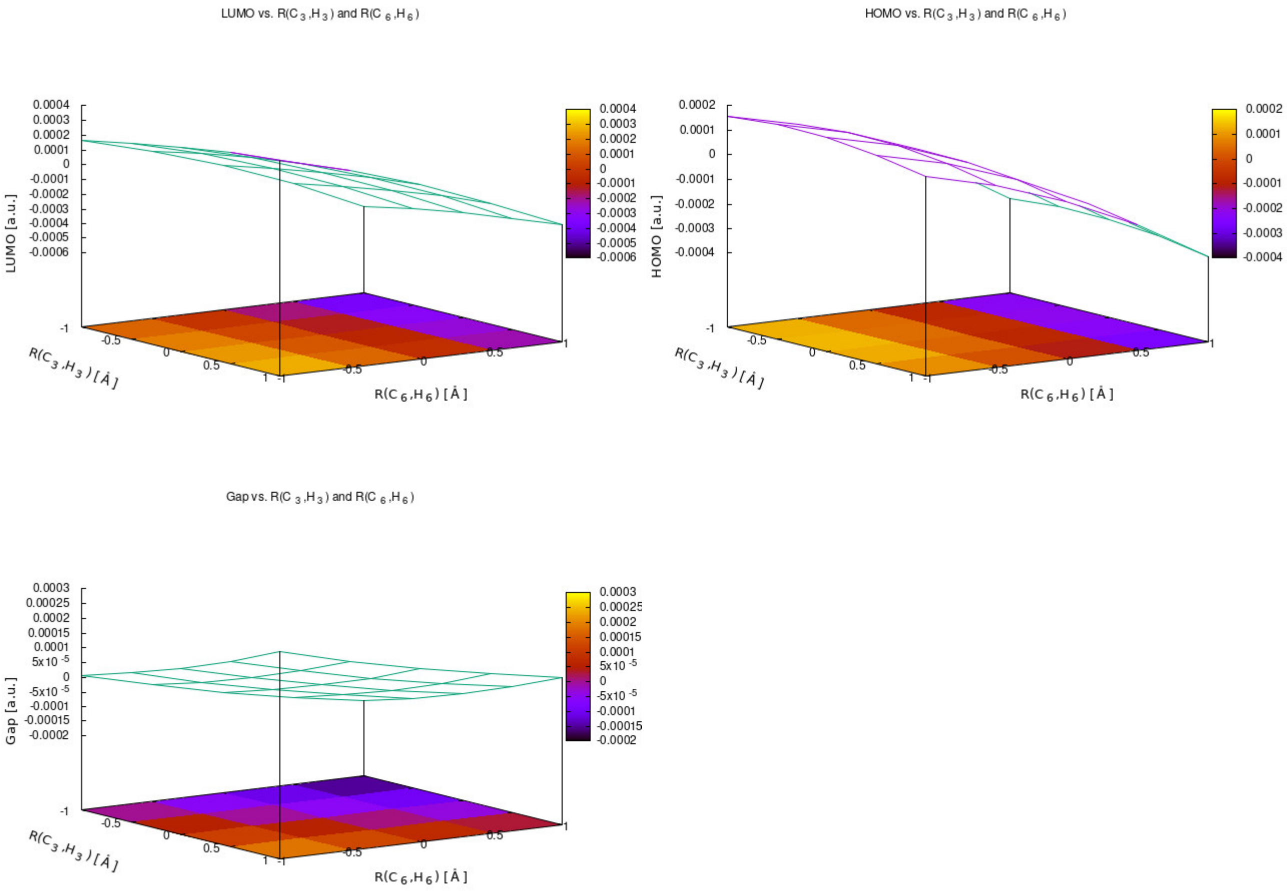

Effect of C_1–N_1 Bond Deformation

Deforming the C_1–N_1 bond (connecting the donor NH_2 to the aromatic ring) induces small orbital variations. Figure S5 shows changes in HOMO, LUMO, and HOMO–LUMO gap under symmetric stretching/contraction (±0.1 Å). The changes are < 0.01 a.u., about one-tenth of computational-level effects.

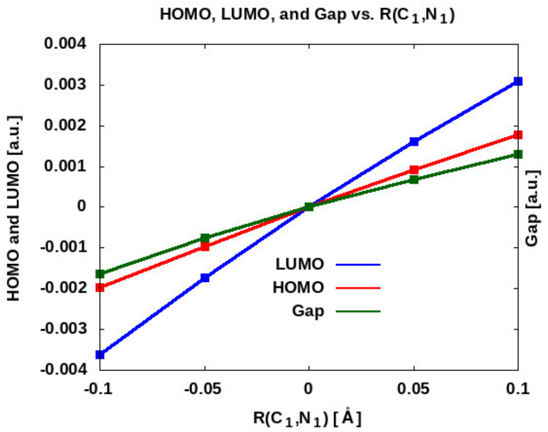

Figure S5: HOMO (center), LUMO (left), and HOMO–LUMO gap (right) energies of pNA relative to gas-phase equilibrium [a.u.] as a function of C_1–N_1 bond stretching/contraction (±0.1 Å).

Coupled Torsional and Vibrational Effects

A 90° NH_2 rotation combined with H_3 contraction or stretching (±0.1 Å) produces nearly identical orbital profiles, with total variations ∼0.025 a.u., showing that superimposed vibrations are minor.

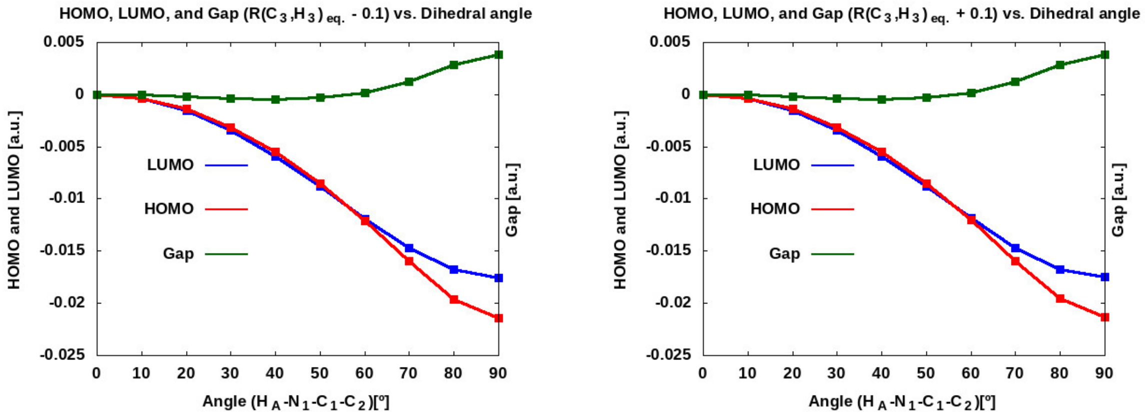

Figure S6: HOMO, LUMO, and HOMO–LUMO gap [a.u.] for pNA with H_3 contracted (left) or stretched (right) by 0.1 Å, as a function of dihedral H_A–N_1–C_1–C_2.

Summary of Localized Deformation Effects

- Dipole moment variations ≤ 0.05 a.u.;
- Frontier orbital changes ≤ 0.03 a.u.;
- Effects on α, β, and γ negligible compared to torsional motion.

Localized vibrations provide only minor contributions compared to torsional deformations.

Localized and Coupled Effects on Polarizability α

While torsional deformations dominate the variation of α, localized bond and vibrational effects also produce systematic trends. Here we present the main results for individual hydrogen stretching, combined H_3/H_6 displacement, C_1–N_1 bond deformation, and torsion–vibration coupling.

Individual and Combined Hydrogen Stretching

Figure S7 shows the impact of stretching H_3 (left) and H_2 (right) on α, relative to the gas-phase equilibrium value. Both cases display a slightly concave monotonic increase with elongation, with H_2 contributing up to ∼1.8 a.u. The combined displacement of H_3 and H_6 leads to a total variation of ∼3.0 a.u., approximately the sum of the individual contributions.

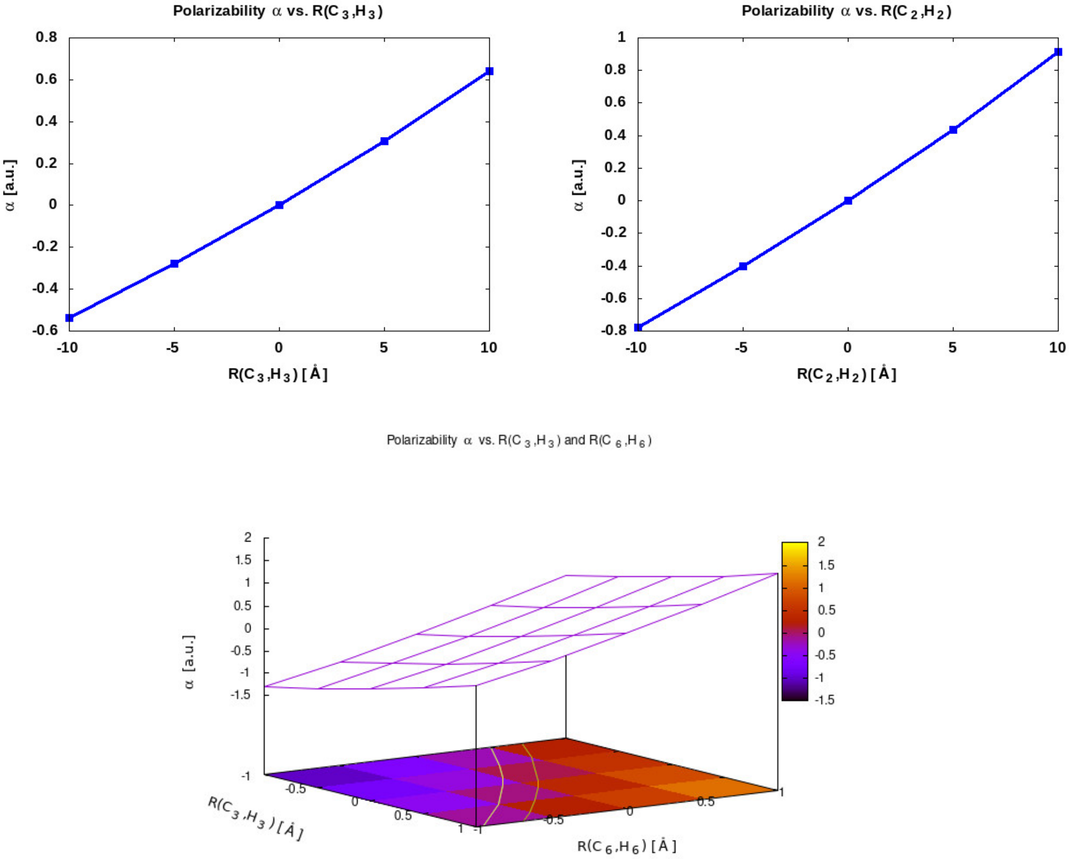

Figure S7: Polarizability α [a.u.] as a function of symmetric stretching/contraction (±0.1 Å) for H_3 (top left), H_2 (top right), and combined H_3/H_6 displacement (bottom).

C_1–N_1 Bond Deformation

The variation of α with C_1–N_1 bond deformation is shown in Figure S8. A monotonic decrease occurs with bond shortening, producing a total change of ∼1.4 a.u., comparable to the individual H_2 stretching effect but in the opposite direction.

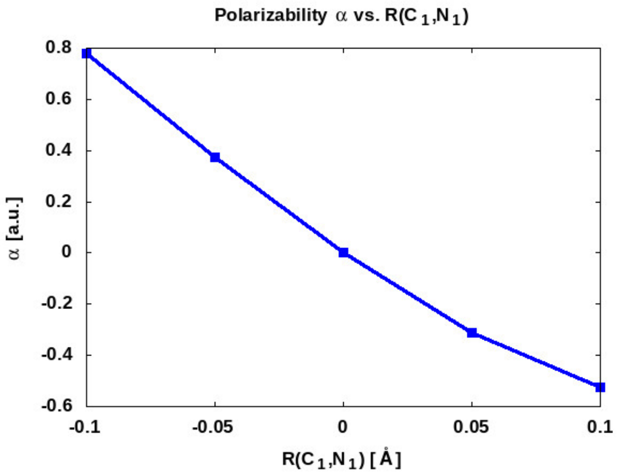

Figure S8: Polarizability α [a.u.] as a function of symmetric stretching/contraction (±0.1 Å) of the C_1–N_1 bond.

Torsion–Vibration Coupling

To examine the effect of combined torsional and vibrational motions, we considered a 90° rotation of the NH_2 group together with H_3 stretching (right) or contraction (left) by 0.1 Å. Figure S9 shows that the resulting variations in α are ∼2.5 a.u., roughly one-third of the pure torsional effect. This confirms that localized vibrations add only minor contributions to the polarizability under torsional distortion.

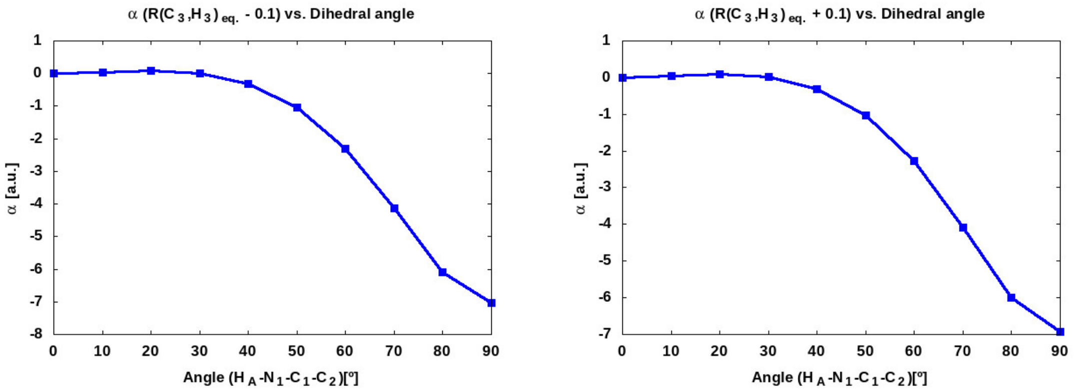

Figure S9: Polarizability α [a.u.] for NH_2 rotation (90°) combined with H_3 contraction (left) or stretching (right) by 0.1 Å. Vibrational effects provide only minor additional variations.

Summary of Polarizability Response

Across all localized and coupled deformations:

- α varies by ≤ 3 a.u. for local hydrogen and bond displacements;
- torsional motion remains the dominant factor, producing up to ∼8.5 a.u. change;
- coupled torsion–vibration effects add only minor contributions.

These results confirm that polarizability is primarily driven by large-amplitude torsional deformations, while localized vibrations have a limited influence, in agreement with the conclusions of the main text.

Localized and Coupled Effects on β

While torsional deformations dominate the variation in β, localized vibrations and bond distortions also produce measurable but smaller effects.

Hydrogen Stretching

Figure S10 shows the impact of H_3 (left) and H_2 (right) stretching on β. For H_3, both β^{J_1} and β^{J_3} decrease monotonically, with a total variation of $\sim 2 \times 10^5$ a.u. H_2 stretching shows a different behavior: the octupolar term increases monotonically, while the dipolar term decreases under elongation by $\sim 6 \times 10^4$ a.u. These changes are roughly one order of magnitude smaller than those induced by group rotations.

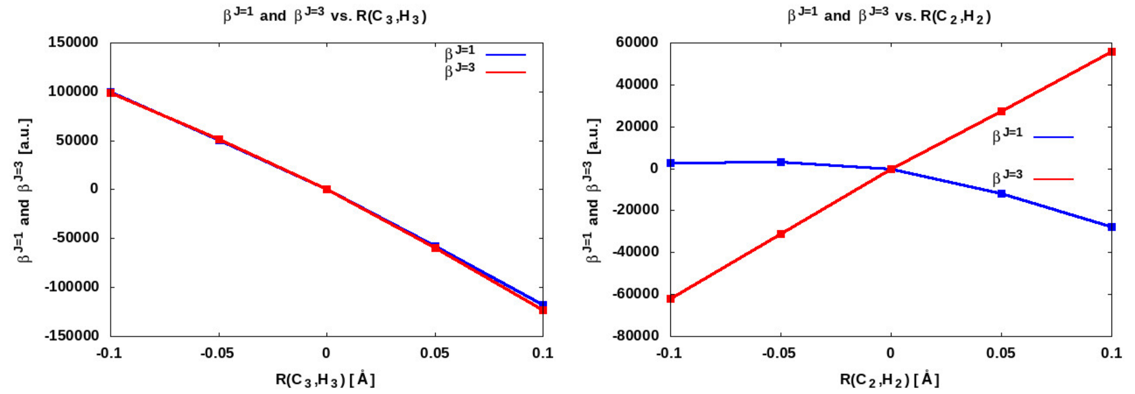

Figure S10: Hyperpolarizability β [a.u.] as a function of symmetric stretching/contraction (±0.1 Å) for H_3 (left) and H_2 (right).

Combined Hydrogen Displacement

Extending to combined motions, Figure S11 shows the simultaneous stretching of H_3 and H_6. β^{J_1} reaches its maximum when both bonds are contracted and minimum when both are stretched, while β^{J_3} displays a 90° symmetry, swapping maxima and minima. Despite the complex surfaces, total variations remain $\sim$ one-tenth of the change induced by altering the computational level.

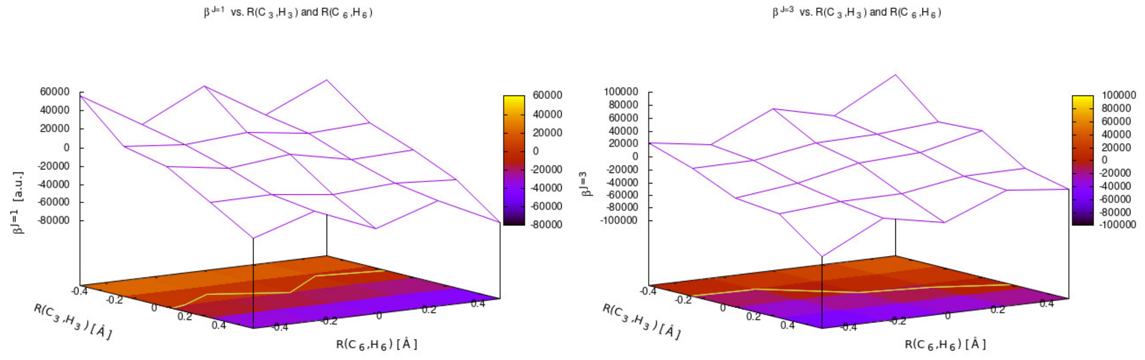

Figure S11: Hyperpolarizability β [a.u.] as a function of combined H_3/H_6 stretching/contraction (±0.1 Å): β^{J_1} (left) and β^{J_3} (right).

C_1–N_1 Bond Deformation

The response of β to C_1–N_1 bond deformation is shown in Figure S12. β^{J_1} and β^{J_3} vary by $\sim 1.6 \times 10^6$ and $\sim 1.0 \times 10^6$ a.u., respectively. Both components increase with elongation up to 0.05 Å and decrease beyond this point, with the curves crossing at the equilibrium bond length. These variations remain smaller than those induced by torsional rotation.

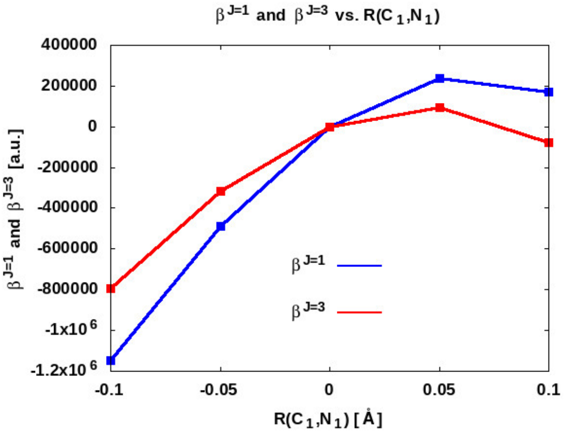

Figure S12: Hyperpolarizability β [a.u.] as a function of symmetric stretching/contraction (±0.1 Å) of the C_1–N_1 bond.

Torsion of β^{J_1} and β^{J_3} components

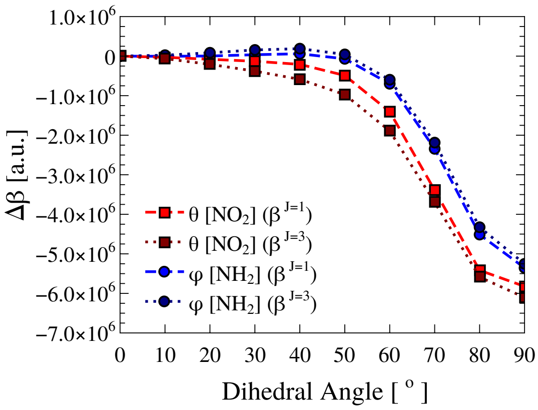

Figure S13: Relative first hyperpolarizability ($\Delta\beta = \beta - \beta_{\text{gas}}$, [a.u.]) of pNA as a function of the donor torsion φ (NH_2, blue) and the acceptor torsion θ (NO_2, red). The reference point $(\varphi, \theta) = (0°, 0°)$ corresponds to the equilibrium gas-phase geometry with $\Delta\beta = 0$. Both torsions show threshold-dependent responses: β remains nearly constant at low angles and decreases abruptly beyond 40–50°, dominated by octupolar contributions. Results are shown separately for the $J = 1$ and $J = 3$ tensorial components. The reference first hyperpolarizability components at equilibrium are $\beta_1 = 5.7 \times 10^6$ a.u. and $\beta_3 = 6.1 \times 10^6$ a.u., respectively.

Torsion couping of β^{J_1} and β^{J_3} components

Torsion–Vibration Coupling

Figure S15 shows the hyperpolarizability β concerning the gas phase equilibrium magnitude due to the rotation in 90° of the NH_2 group when the H_3 bond is contracted 0.1Å(left) and when it is stretched 0.1Å(right). The results confirm that vibrational perturbations have a minor additional effect.

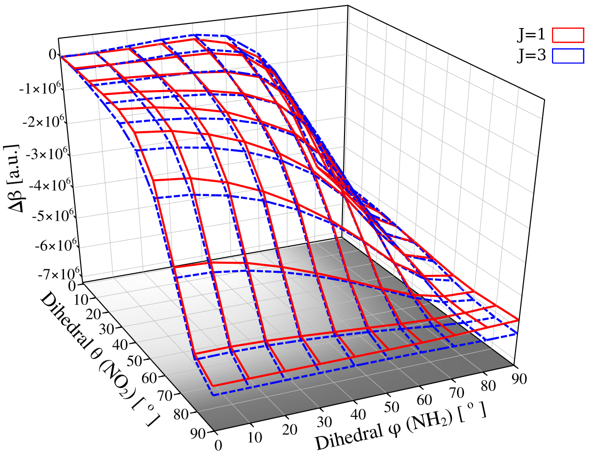

Figure S14: Relative first hyperpolarizability ($\Delta\beta = \beta - \beta_{\text{gas}}$, [a.u.]) of pNA as a function of the dihedrals φ (NH_2) and θ (NO_2). Red solid wireframe: $J = 1$; blue dashed wireframe: $J = 3$. The reference $(\varphi, \theta) = (0°, 0°)$ corresponds to the equilibrium gas-phase geometry with $\Delta\beta = 0$. Both components decrease monotonically with torsion. The reference first hyperpolarizability components at equilibrium are $\beta_1 = 5.7 \times 10^6$ a.u. and $\beta_3 = 6.1 \times 10^6$ a.u., respectively.

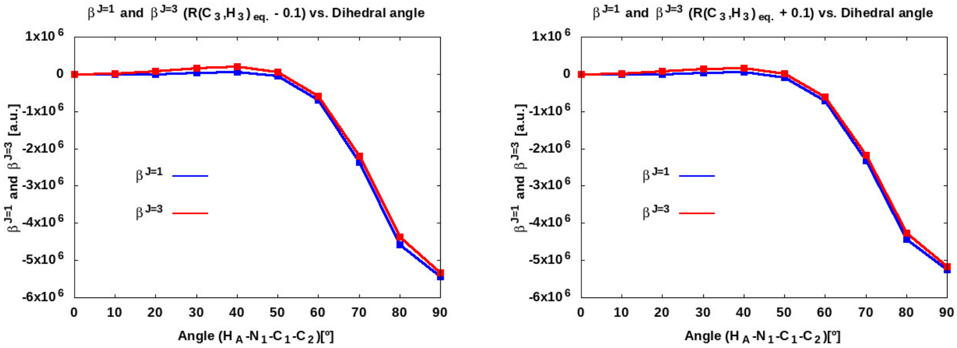

Figure S15: Hyperpolarizability β [a.u.] for NH_2 rotation (90°) combined with H_3 contraction (left) or stretching (right) by 0.1 Å.

Summary of β Response

Across all localized and coupled deformations:

- Torsional rotation dominates, inducing ~ 5–6×10^6 a.u. changes;
- Local H and C_1–N_1 bond displacements produce variations $\leq 10^6$ a.u.;
- Torsion–vibration coupling adds negligible additional impact.

These results confirm that the first hyperpolarizability of pNA is primarily modulated by large-amplitude torsional motions, while localized vibrations have minor influence.

Localized and Coupled Effects on γ

While γ exhibits sharp resonances under torsional motion, localized bond and vibrational deformations produce smaller, quasi-linear responses.

Individual Hydrogen Stretching

Figure S16 shows the effect of symmetric stretching of H_3 (left) and H_2 (right). Elongation of H_3 decreases γ, while elongation of H_2 increases it, mirroring the opposing electronic effects of donor vs. acceptor regions. The magnitudes remain far below those induced by torsional rotation.

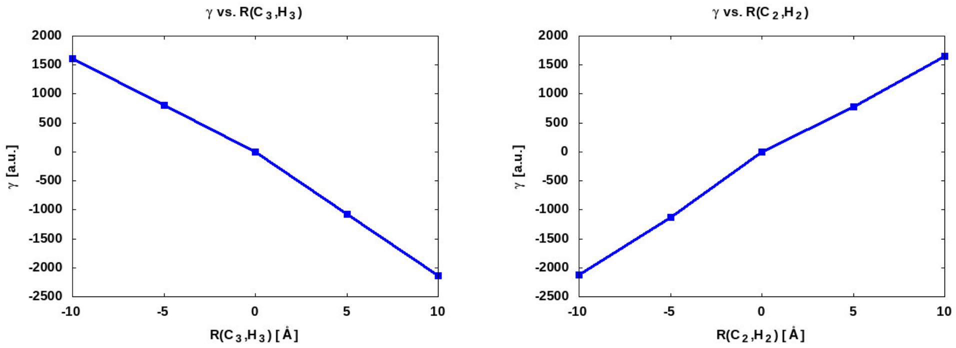

Figure S16: Hyperpolarizability γ [a.u.] as a function of symmetric stretching/contraction (± 0.1 Å) for H_3 (left) and H_2 (right).

Combined Hydrogen Displacements

Figure S17 shows the combined effect of H_3/H_6 stretching. Elongating H_3 decreases γ, while elongating H_6 increases it, producing a response surface that reflects the superposition of these opposing effects.

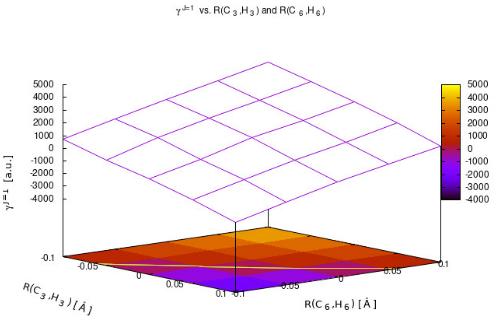

Figure S17: Hyperpolarizability γ [a.u.] as a function of combined H_3/H_6 stretching/contraction (± 0.1 Å).

C_1–N_1 Bond Deformation

Figure S18 presents the variation of γ under C_1–N_1 bond deformation. A monotonic increase is observed with bond elongation, reaching $\sim 2.5 \times 10^5$ a.u., with a stronger response to elongation than contraction.

Torsion–Vibration Coupling

To complete the analysis, Figure S20 shows γ under a 90° NH_2 rotation combined with H_3 contraction (left) or stretching (right). The curves remain similar to the isolated rotation case, with the resonance near 75° still present.

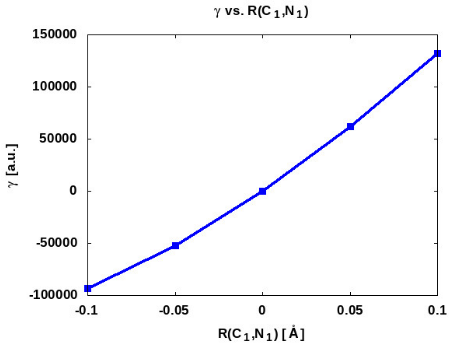

Figure S18: Hyperpolarizability γ [a.u.] as a function of symmetric stretching/contraction ($\pm$0.1 Å) of the C_1–N_1 bond.

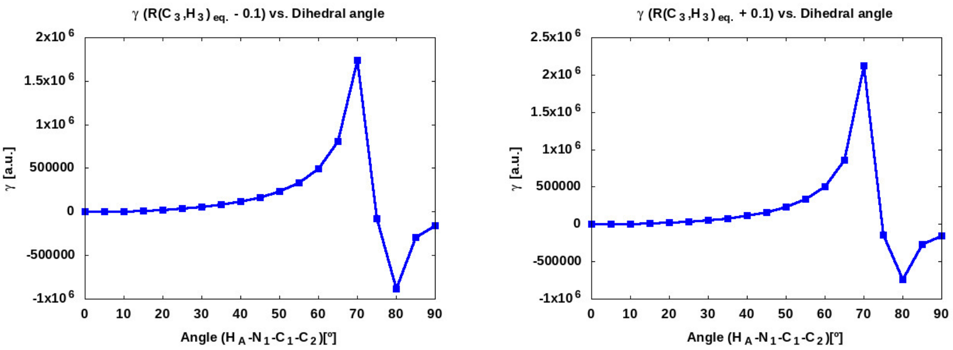

Figure S19: Hyperpolarizability γ [a.u.] for NH_2 rotation (90°) combined with H_3 contraction (left) or stretching (right) by 0.1 Å.

Summary of γ Response

- Torsional resonances dominate the γ response (near 75° for NH_2 and 35° for NO_2);
- Local bond and H-stretch deformations induce variations $\ll 10^6$ a.u.;
- Coupled torsion–vibration effects produce only minor additional influence.

Overall, γ is primarily modulated by large-amplitude torsional motion, while localized vibrations have negligible impact on its value.

Overall Summary of Structural Effects

Overall, the analysis confirms that torsional deformations of the NH_2 and NO2 groups dominate the NLO response of pNA, inducing variations up to an order of magnitude larger than those caused by local H-atom or C1–N1 bond distortions.

Overall, the analysis confirms that torsional deformations of the NH_2 and NO_2 groups are the dominant factor governing the NLO response of pNA, leading to variations up to an order of magnitude larger than those caused by local H or C_1–N_1 bond distortions. Coupled torsion–vibration effects remain minor and do not qualitatively modify the response surfaces. The resulting sensitivity hierarchy is clear: dipole $< \alpha < \beta < \gamma$.

IR Spectrum

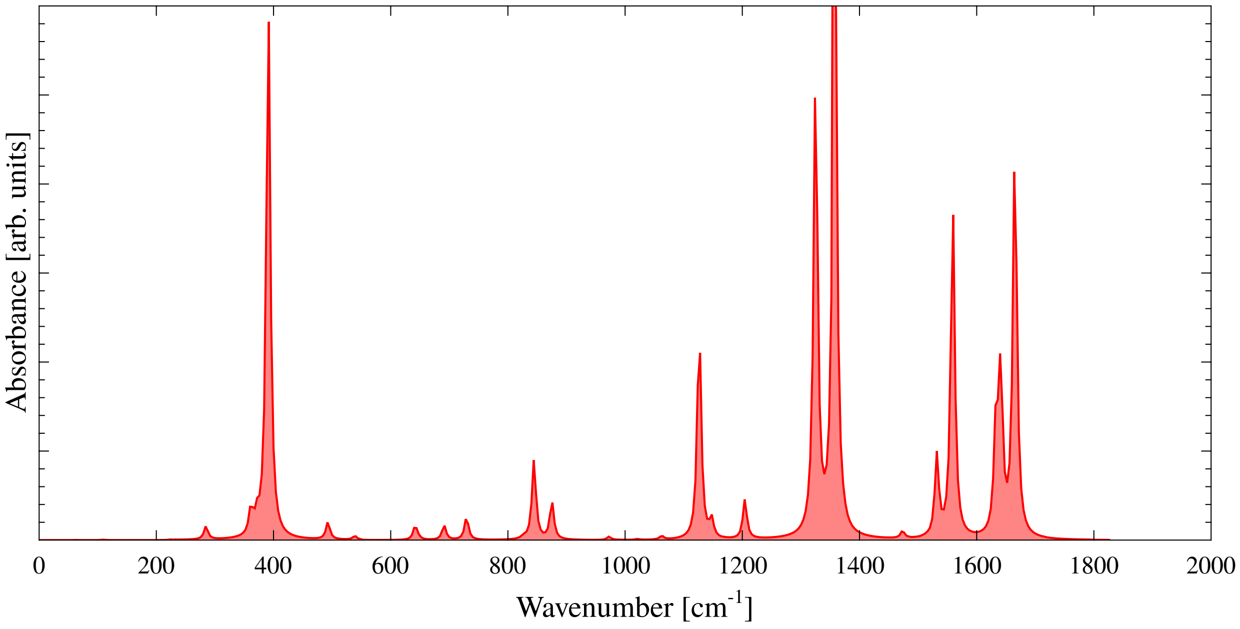

Figure S20: Computed IR spectrum of pNA. Intensities are given in arbitrary units and have been scaled for clarity.

Table S2: Computed harmonic vibrational frequencies and IR intensities of *p*-nitroaniline at B3LYP/6-311++G** level of theory. The lowest-frequency torsional modes correspond to the donor and acceptor rotations: Mode 1 (63 cm^{-1}) is mainly the NO_2 torsion (θ), and Mode 6 (371 cm^{-1}) is associated with the NH_2 torsion (φ). Although these torsional modes exhibit weak IR intensities, their low frequencies make them thermally accessible and thus dynamically relevant for modulating conjugation and donor–acceptor charge transfer. All other modes are listed for completeness.

Mode	Frequency (cm^{-1})	IR Intensity (km mol^{-1})
1	63.19	0.0695
2	109.24	0.2901
3	224.80	0.3939
4	284.77	8.6587
5	361.40	16.6444
6	371.63	10.7181
7	391.14	351.4261
8	393.95	0.1940
9	424.11	0.0004
10	492.88	11.4419
11	538.71	2.4030
12	641.85	9.3579
13	648.13	9.9019
14	691.05	9.6110
15	729.37	14.9837
16	817.65	0.0584
17	826.69	1.4944
18	844.52	52.3125
19	875.01	24.9698
20	972.51	2.0670
21	983.57	0.0038
22	1020.78	0.5249
23	1062.41	2.3245
24	1126.48	138.6982
25	1147.59	11.9912
26	1204.42	25.6852
27	1325.16	304.7066
28	1332.53	0.0000
29	1357.68	454.8470
30	1372.52	1.9237
31	1473.50	4.7129
32	1532.03	52.8284
33	1559.24	216.9544
34	1633.06	70.9350
35	1641.07	103.5920
36	1665.25	257.7747
37	3170.64	8.9822
38	3171.30	11.5414
39	3219.89	0.4417
40	3220.01	3.9432
41	3594.55	76.9666
42	3701.76	30.9539
